# Theory-Guided Discovery of Ion-Exchanged Poly(heptazine imide) Photocatalysts Using First-Principles Many-Body Perturbation Theory

**DOI:** 10.1021/jacs.5c09930

**Published:** 2026-01-07

**Authors:** Zahra Hajiahmadi, Anna Lo Presti, S. Shahab Naghavi, Markus Antonietti, Christian Mark Pelicano, Thomas D. Kühne

**Affiliations:** † CASUS - Center for Advanced Systems Understanding, 28414 Helmholtz-Zentrum Dresden-Rossendorf E.V. (HZDR), Untermarkt 20, Görlitz D-02826, Germany; ‡ Department of Colloid Chemistry, 28321 Max Planck Institute of Colloids and Interfaces, Potsdam D-14476, Germany; § Department of Physical and Computational Chemistry, 426068 Shahid Beheshti University, Tehran 1983969411, Iran

## Abstract

Poly­(heptazine imides) (PHI) show strong promise in photocatalysis, but limited control over electronic properties continues to constrain their full potential. We modulated PHI’s photocatalytic activity to overcome this limitation by incorporating mono-, di-, and trivalent metal cations into its framework. We employed calculations based on many-body perturbation theory—a highly accurate approach for electronic structure calculations—which provides improved accuracy in quasiparticle energy predictions compared to conventional density functional theory, particularly for band gaps and excitonic properties, to elucidate the underlying mechanisms. The coupling between exchanged metals and the resulting optoelectronic properties is often nontrivial: Pd, Pt, and Cu^1+^, for example, produce favorable band structures but show limited photocatalytic activity due to optically forbidden intra-atomic d–d transitions. Our analysis identified several metal-doped PHI systems with electronic structures well suited for hydrogen and oxygen evolution, CO_2_ reduction, and H_2_O_2_ production. Guided by these theoretical insights, we synthesized a subset of *M*-PHI materials (where *M* is either K, Na, Li, Ca, Mg, or Zn) predicted to enhance photocatalytic reactivity. Photocatalytic measurements confirm substantial increases in H_2_O_2_ generation rates—up to 4.5-fold higher than undoped PHI—for these candidates, underscoring the effectiveness of our design strategy. These findings offer molecular-level insights into tailoring *M*-PHI interactions, paving the way for next-generation photocatalysts.

## Graphical abstract

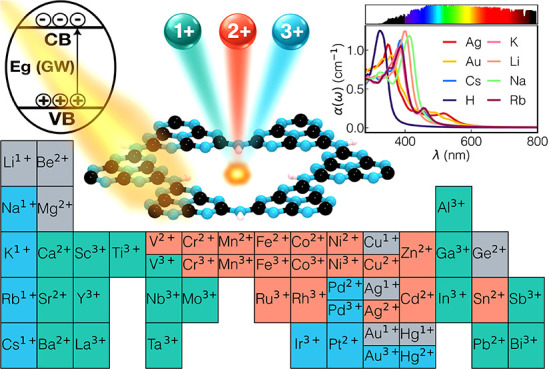

## Introduction

Photocatalytic semiconductors offer a sustainable approach to addressing the global energy crisis and environmental challenges by converting solar energy into value-added chemical fuels.^1^ Among these, carbon nitride-based materials have emerged as promising candidates due to their low cost, nontoxicity, thermal stability, and tunable electronic structures.^2–4^ The term g-C_3_N_4_ refers to a broad family of layered materials built from triazine or heptazine units. Its layered structure and the presence of an extended π-conjugated framework enhance charge mobility and suppress bulk recombination.^5^ Unlike inorganic photocatalysts, which often possess fixed and wide band gaps that limit them to the ultraviolet region,^6,7^ g-C_3_N_4_ exhibits a relatively narrow band gap (≈2.7 eV), allowing visible light absorption.^8^ However, early g-C_3_N_4_ photocatalysts often suffer from a limited structural order and high exciton binding energies, which hinder charge separation and transport.^2,9,10^

Metal poly­(heptazine imide) (*M*-PHI), a structurally ordered derivative of g-C_3_N_4_, has recently gained attention for diverse photocatalytic applications,^11,12^ such as hydrogen and oxygen evolution reaction (HER and OER),^13,14^ H_2_O_2_ generation (oxygen reduction reaction (ORR)),^15^ and CO_2_ reduction.^16^ Compared to conventional covalent carbon nitride, *M*-PHI exhibits improved crystallinity, charge mobility, and porosity, which together enhance light absorption and charge separation.^17^ These advantages are reflected in their performance; *M*-PHIs, for instance, often deliver H_2_ evolution rates 10–100 times higher than melon-like carbon nitrides under the same conditions.^18^

Metal cations in *M*-PHI coordinate with negatively charged nitrogen atoms on the imide bridges surrounding the pores. These anionic sites not only provide a high surface area for reactant adsorption, but also facilitate a uniform distribution of diverse metal cations, enhancing conductivity and catalytic activity.^19,20^

Cation exchange is typically carried out by immersing the *M*-PHI framework in aqueous metal salt solutions. This method offers a simple, scalable, rapid, and structure-preserving route to metal functionalization.^17^ The resulting interlayer and pore architectures vary depending on the nature and hydration radius of the cations exchanged.^10^ This ion exchange approach presents a notable advantage over more complex methods, such as direct thermal polymerization with metal salts, by offering a simpler and more adaptable route to obtain a broad range of metal-functionalized PHI materials.

A variety of *M*-PHI catalysts containing alkali metals (K, Li, and Na), alkaline earth metals (Mg, Ca, and Ba), and transition metals (Zn, Mn, Ru, Fe, Ni, and Co) have been explored for OER and HER, respectively, to date.^10,21,22^ However, transition metals such as Fe, Ni, Co, and Ru show limited activity for ORR.^16^ Although numerous cations have been studied,^23–28^ a systematic and holistic investigation aimed at identifying optimal species for specific photocatalytic applications is still lacking.

In order to address this gap, we present a comprehensive computational investigation of a wide range of cations (*M* ^+^, *M* ^2+^, and *M* ^3+^), hosted within the PHI framework. We focus on how these cations influence the structural, electronic, and optical properties of the material. Key descriptors, including bonding interactions, charge distribution, band gap and alignment, exciton binding energy, and charge mobility, are elevated to guide the photocatalyst selection.

Photocatalytic processes arise from excited-state processes; therefore, *M*-PHI systems are not adequately described by ground-state density functional theory (DFT) alone. In particular, standard DFT often fails to capture electron excitation and correlation effects accurately.^29^ We employed the many-body perturbation theory within the GW approximation, where G is the Green’s function and W is the screened Coulomb interaction, to remedy this deficiency. The GW method accounts for quasiparticle effects and systematically reduces any self-interaction errors inherent in DFT, thereby providing a more reliable description of the electronic structure and band gaps.^30–32^

Based on these GW-derived results, we defined four screening criteria for high-performance *M*-PHI photocatalysts: (i) a suitable band gap (>1.25 eV) for visible-light absorption, (ii) band edge positions aligned with redox potentials of relevant reactions (e.g., HER, OER, ORR, and CO_2_ reduction), (iii) delocalized states near band edges as indicated by the projected density of states (PDOS), and (iv) optical properties. These criteria reflect fundamental photocatalytic design principles emphasizing light harvesting, charge separation, and redox capability. Our study offers a predictive and transferable framework for rational photocatalyst design. Finally, some selected materials were successfully synthesized and experimentally validated, demonstrating the enhanced H_2_O_2_ production activity and confirming the practical relevance of our computational framework.

## Results and Discussion

### Structure and Bonding

In order to understand how metal cations influence the photocatalytic activity of PHI, we began with the pristine, layered bulk structure of PHI as a reference. The PHI consist of heptazine units covalently bonded through sp^2^-hybridized C–N and N–H bonds. Each layer has a macrocycle pore characterized by two orthogonal distances (*d*_1_ = 12.94, *d*_2_ = 10.28 Å), offering potential sites for guest ions (see Figure 1). The layers stack via van der Waals interactions, with an interlayer spacing of 4.66 Å. We substituted nitrogen-bonded hydrogen atoms with metal cations to construct *M*-PHI systems: three H atoms with three *M* ^+^, two H atoms with one *M* ^2+^, and three H atoms with one *M* ^3+^.

**Figure 1:**
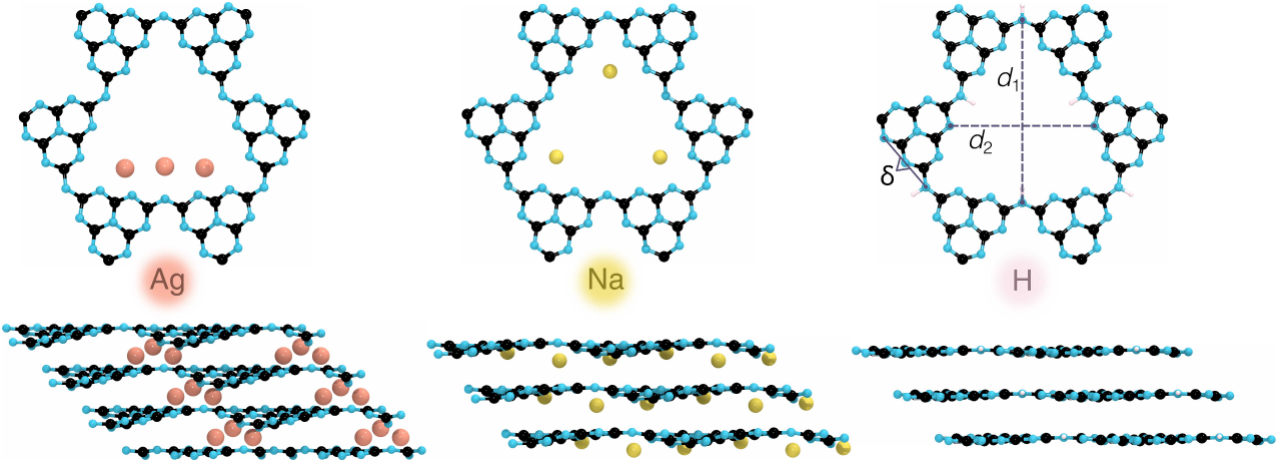
Top and side views of Ag­(I)-PHI, Na-PHI, and H-PHI (from left to right), shown as representative examples of cation locations and structural distortions. In H-PHI, *d*_1_, *d*_2_, and δ indicate the distortion indices reported in Table S1.

After full structural relaxation, the cations remain either within the PHI plane (inside the pores) or intercalate between the layers, inducing lattice expansion or structural distortion, as shown in Figures 1 and S1–S3. The thermodynamic stability of these structures is supported by ab initio molecular dynamics simulations using Ag-PHI and Na-PHI as representative examples (Figure S4), as well as by the predominantly negative enthalpies of formation for most structures listed in Tables S2–S4.

The pore geometry in the distorted configuration changes, while the polymer backbone remains intact. We quantified these structural responses by measuring the angle (δ) between three nitrogen atoms and tracking variations in the pore dimensions (*d*_1_, *d*_2_). Table S1 reveals that structural relaxation leads to cation positioning either in-plane or in the interlayer space, accompanied by distinct geometric distortions of the PHI framework.

We categorized all structures into four groups based on their distortion level and cation’s position (in-plane or interlayer). Taking pristine H-PHI (δ = 177.58^◦^), as the reference, we set the distortion threshold and labeled structures with δ < 175^◦^ as distorted. The classification shows that distortion and site preference depend on the cation’s size^33^ and electronegativity (EN).^34^ The charge density difference (CDD) from the Bader analysis reinforces this trend by tracking how each cation’s charge shifts from its isolated state to its incorporated state in H-PHI, as shown in Figure 2. Larger cations (atomic radius *r* > 170 pm) tend to expand the lattice and remain in-plane; those with large CDD values (CDD > 1.5) distort the framework significantly. By contrast, smaller cations are typically located between layers. Among these, those with low CDD values (0.4–0.5) induce noticeable distortions, while those with intermediate CDD values (0.9–1.3) do not produce any appreciable structural changes. A comprehensive summary of size, EN values, and the corresponding distorted parameters is provided in Table S1. It is worth noting that three bulky monovalent (1+) cations are needed to fill each pore, and their combined steric effect induces greater lattice change than a single 2+ or 3+ cation. The nature of the cation sets the distortion and its position within its bonds with its surroundings, and this interaction shapes the system’s conductivity^10^ and charge mobility,^28^ as discussed in the following sections.

**Figure 2:**
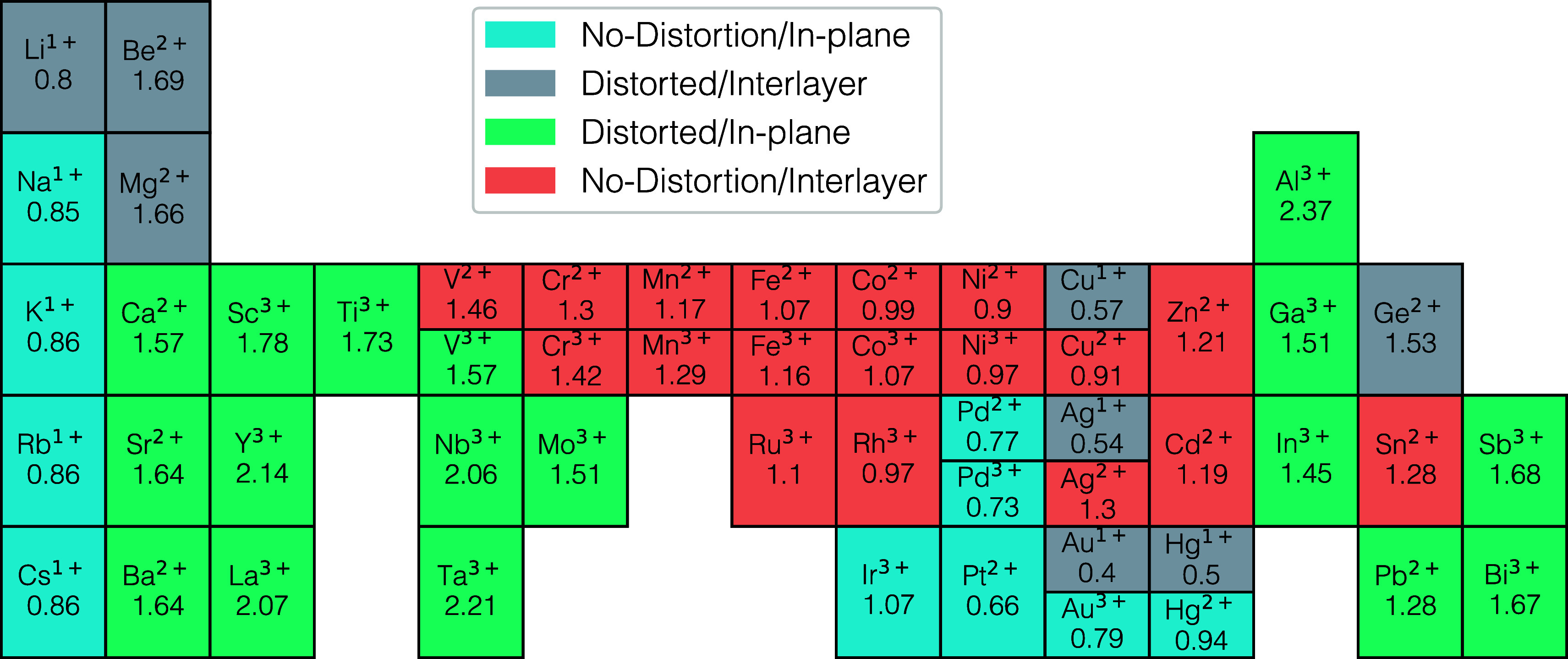
Charge density difference (CDD) values of mono-, di-, and trivalent metal cations classify structural changes in *M*-PHI: No-Distortion/In-plane (blue), No-Distortion/Interlayer (salmon pink), Distorted/In-plane (green), and Distorted/Interlayer (gray).

Hydrogen atoms form polar covalent bonds with nitrogen in pristine PHI. Metal substitution alters this landscape, as shown by the integrated crystal orbital bond index (ICOBI) and integrated crystal orbital Hamilton population index (ICOHP), both of which are reliable indicators of the bond character.^35^ The ICOBI and −ICOHP values are reported in Tables 1 and S2–S4, respectively. The COHP and its integration up to the Fermi level (ICOHP) reveal the character and strength of each bond. Since the bond order equals the number of electrons in bonding states minus those in antibonding states (negative values), a larger integrated ICOHP indicates stronger bonding. Complementing this, the ICOBI values measure the electron sharing between nearest atoms, ranging from zero (fully ionic) to one and above (covalent) bonds, providing an alternative perspective on chemical bonding.^35,36^ Alkali metals, with larger −ICOHP and negligible ICOBI (≈10^–2^), form purely ionic bonds with nitrogen atoms that position at the triangular pore corners (Figure 1). The aforementioned quantitative analysis is visualized in the CDD (Δρ) plots of (Figures 3-(bottom), S5 and S6), which show that alkali metals transfer nearly all their charge to nitrogen, forming predominantly ionic bonds, while transition and coinage metals share electrons partially with nitrogen and, in some cases, with each other, producing a continuum of bonding from ionic to covalent that shapes the electronic structure and charge mobility of *M*-PHI. Unlike alkali metals, which sit at the three corners of the pore and interact primarily through ionic forces, coinage metals—silver, copper, and gold—approach each other more closely. Previous studies have shown that coinage metals can form metal–metal bonds even within crystalline systems, reflecting their intrinsic tendency for such interactions.^37–39^ This metallic interaction stems from the relatively higher EN of transition metals, which also shifts their electronic states toward the valence band edge, making them active contributors to the electronic structure of *M*-PHI. As Table 1 shows, C–N bonds retain a strong covalent character, with high ICOBI and ICOHP values, preserving the framework under chemically aggressive conditions, as confirmed by high-temperature molecular dynamics simulations (Figure S4). This hierarchy of bonding—ranging from ionic alkali metals to metallic coinage metals interactions—directly influences the electronic structure of *M*-PHI. Understanding these bonding patterns is essential for predicting how metal substitution affects bands edges, charge distribution, and, ultimately, the material’s suitability as a photocatalyst.

**Table 1:** Values of −ICOHP (eV), ICOBI (eV), and CDD for C–N, N–M, C–M, (M=Metal Cations)

		C–N	N–M	C–M	M
H-PHI	–ICOHP	12.36	0.05	0.080	
	ICOBI	1.14	0.82	0.002	
Na-PHI	–ICOHP	13.57	0.38	0.090	
	ICOBI	1.30	0.08	0.007	
	CDD				0.85
Mg-PHI	–ICOHP	13.69	1.05	0.095	
	ICOBI	1.34	0.18	0.006	
	CDD				1.66
Zn-PHI	–ICOHP	13.85	1.66	0.006	
	ICOBI	1.37	0.28	0.005	
	CDD				1.21
Cu(II)-PHI	–ICOHP	13.82	1.81	0.042	
	ICOBI	1.34	0.26	0.004	
	CDD				0.92
Ag(I)-PHI	–ICOHP	13.31	1.57	0.082	
	ICOBI	1.26	0.27	0.009	
	CDD				0.53

**Figure 3:**
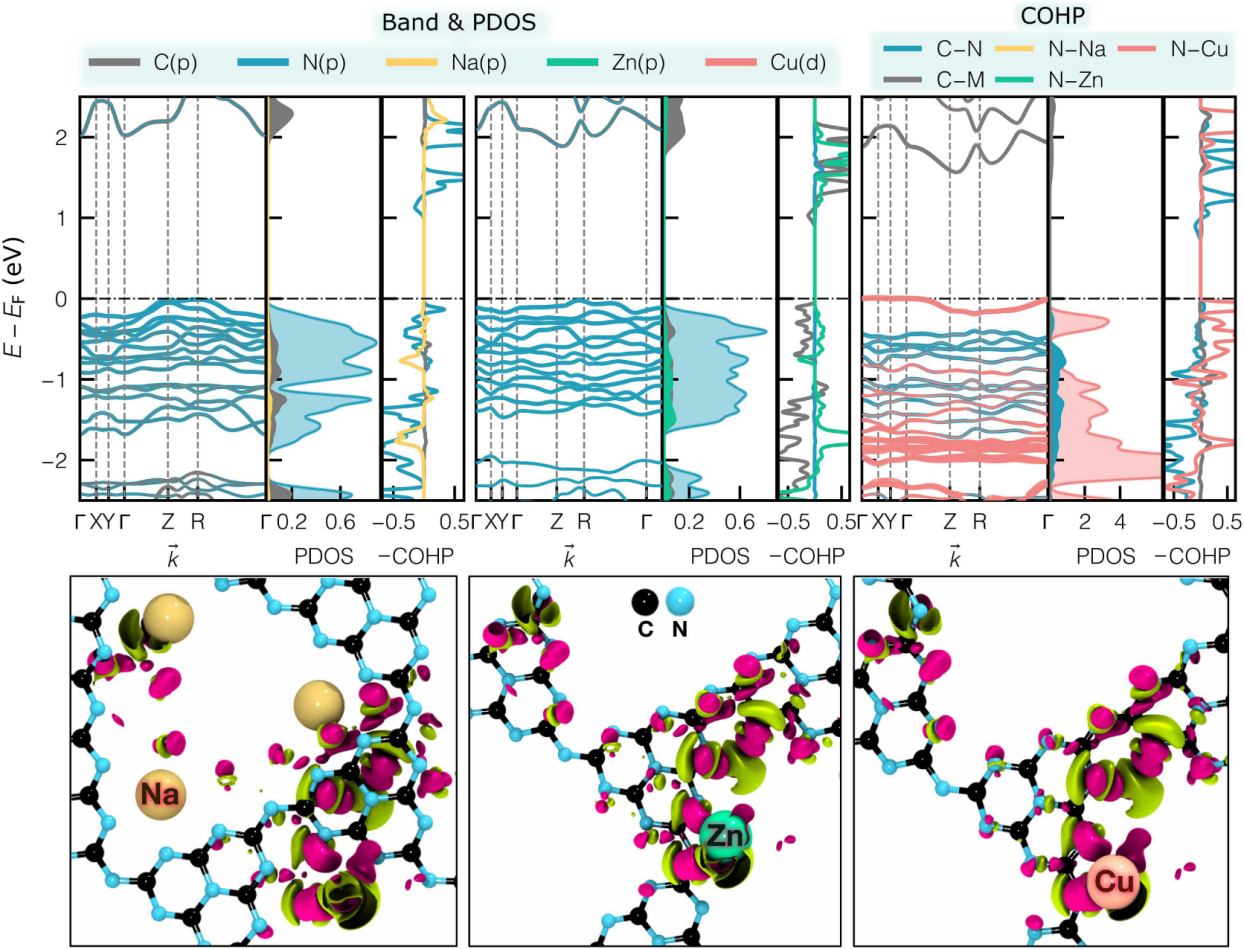
(Top panel): atomic-orbital projected electronic band structures, density of states, and the COHP bond plots; (bottom panel): charge density differences (accumulation in pink and depletion in green) are plotted in ball-and-stick model visualization for Na-PHI, Zn-PHI, and Cu­(II)-PHI, from left to right, respectively (carbon: black, nitrogen: blue, sodium: yellow, zinc: green, copper: pink).

### Electronic Properties

The electronic structure determines a material’s ability to drive photocatalytic reactions. A suitable photocatalyst must not only absorb visible light, requiring a band gap between 1.25 and 3.3 eV, but also align its band edges with the redox potentials of the target reactions at a given pH. These criteria guide the selection of metal-doped PHI systems.

#### Band Gaps

Band gaps were calculated using the PBE, SCAN, and GW methods. Among the 55 possible cations considered for substitution at the hydrogen sites, 27 (using PBE), 25 (using SCAN), and 21 (using GW) metal-doped PHI systems were excluded from band alignment analyses due to their metallic character or narrow band gaps below 1.25 eV. (Table S5). Regarding structures with a proper band gap, the band edge positions were obtained from symmetric, nonpolar (001) slab calculations (8 Å thickness) with a 15 Å vacuum perpendicular to the surface to suppress spurious long-range electrostatic interactions. The electrostatic potential within the vacuum-slab supercell was then calculated and aligned with that of the bulk to obtain the band edge positions.^40^ The PBE functional is known to significantly underestimate fundamental band gaps, while meta-GGA functionals, such as SCAN, offer modest improvements.^41^ However, both remain ground-state methods and are not designed to capture excited-state properties, such as optical absorption. As a result, their predicted band gaps deviate substantially from the experimental values and those obtained from many-body perturbation theory, such as the GW approximation.

The latter offers improved accuracy for calculating quasiparticle energy levels,^42,43^ typically within 0.2–0.3 eV of the experimental values,^44^ and outperforms standard DFT approaches.^45,46^ In this case, certain conclusions derived from DFT were qualitatively inaccurate compared to the experimental data, as reported in previous publications.^45–48^ Given GWs’ improved accuracy in quasiparticle gap predictions, cations once deemed unsuitable under PBE—In^3+^, Ir^3+^, V^3+^, Ta^3+^, and Pd^2+^—now lie within an acceptable range.

#### Band Alignment

Beyond band gap predictions, the GW method systematically improves band edge alignment, providing a more predictive and experimentally consistent view of photocatalytic activity. The band edges must straddle the redox potentials of the relevant half-reactions, the conduction band (CB) must lie above *E*_Redox_, and the valence band (VB) below *E*_Oxidation_. Regarding H_2_O_2_ production, the CB must exceed the O_2_/H_2_O_2_ reduction potential (−5.12 eV) and the VB must lie below the H_2_O/O_2_ oxidation potential (−5.26 eV) at pH = 7. Materials that fail to meet both conditions are inactive for photocatalytic reactions.

Overpotentials are inevitable in aqueous photocatalytic systems due to kinetic barriers associated with interfacial charge-transfer reactions. In order to mitigate their influence on the overall feasibility, we deliberately considered materials with sufficiently large band gaps, ensuring that the conduction and valence edges remain well aligned with the redox potentials, even in the presence of realistic overpotentials. The band alignments of 34 compounds at pH = 7 range—the condition commonly used in experimental photocatalytic tests—for water splitting, H_2_O_2_ production, and CO_2_ reduction are shown in Figure 4. In this analysis, GW band alignments reveal the compounds best suited for each reaction. Figure 4 reveals how their band edges match the corresponding redox and oxidation energies. The most promising *M* ^+^ candidates for H_2_O_2_ production are Ag, H, Hg, K, Li, and Na, and optimal *M* ^2+^ species, including Ba, Be, Ca, Cd, Hg, Mg, Pb, Pt, Pd, Sr, and Zn.

**Figure 4:**
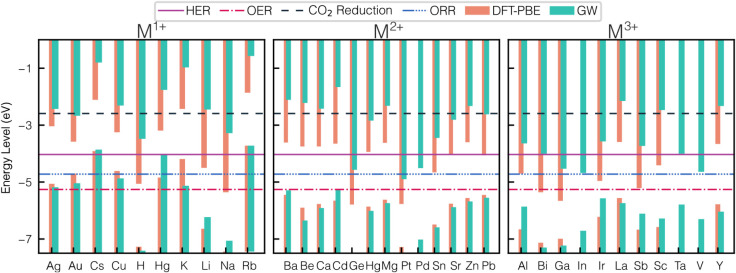
Band alignments of *M*-PHI structures in the PBE and GW method. The HER, OER, ORR, and CO_2_ reduction range in pH = 7 are indicated.

We began by analyzing the structural changes and charge redistribution that result from the incorporation of metal into the PHI framework to identify the key factors regulating band alignment. Although the overall system remains neutral, differences in the charge density between cations and PHI atoms modulate the availability of active sites, thereby promoting reactant adsorption and enhancing the photocatalytic activity. Larger alkali cations (Cs, Rb, K) raise the VB, whereas smaller, more electronegative cations (Ge, Pt, Cu, Sn) lower the VB and shift the CB downward. The Na, whose EN is similar to Rb but differs significantly from Au, was introduced into the relaxed Rb-PHI and Au­(I)-PHI frameworks for our band edge calculation to isolate the effect of EN. Without further structural relaxation (i.e., using a single-point calculation with Na substituted into the Rb- or Au-PHI lattice), this substitution caused no noticeable change in the VB and CB positions (see Table S6), confirming the critical role of geometric distortion in determining electronic properties. Since distortion arises from both the size and charge distribution, these results also highlight their combined influence on the electronic properties of *M*-PHI.

#### Band Edge Delocalization

The projected density of states (PDOS) and band structures (Figures S8 and S9, and 3-(top)) reveal that nitrogen *p*-states dominate the VB of pristine PHI, and carbon *p*-states dominate the CB. Incorporating alkali metals into the PHI framework increases nitrogen’s electronic contribution by transferring charge to and localizing it on nitrogen atoms, as seen in the Δρ plot of Figure 3 (bottom). The Δρ represents the amount of charge transferred upon introducing a cation into the system, assuming that the structure and other parameters remain unchanged. It reflects the net charge transfer originating from the cation and is calculated as

1 $$\Delta \rho =\rho_{M-\mathrm{PHI}}-\rho_{\mathrm{PHI}}-\rho_{M}$$

where ρ_*M*‑PHI_, ρ_PHI_, and ρ_*M*_ denote the charge density of the *M*-PHI, the PHI, and the isolated cations, respectively. These cation-induced partial charges in the PHI system can generate active sites for adsorbing photocatalytically relevant reactants (e.g., H_2_O, OOH^+^, OH^–^).

The VB and CB are largely unaffected by the cations in metals with fully filled *d*-orbitals (e.g., Zn, Cd, and Hg). By contrast, transition metals with partially filled *d*-orbitals perturb both nitrogen and carbon states and contribute significantly to the VB states. Such a control over the VB is a unique characteristic of transition metals with partially filled *d*-states. While optical excitation typically occurs within PHI from N (p) to C (p) for most structures, in some cases, such as Cu^1+^, Ir, Ta, Pd, and Pt, the VB and CB of the metal lie, respectively, higher and lower than those of PHI. As a result, the transition from the VBM to the CBM is intra-atomic, which suppresses optical absorption and renders these systems unsuitable for photocatalytic applications (see Figures S8 and S9).^49,50^

Beyond these cases, there is another subset of dopants that disqualifies the compounds through their electronic character near the Fermi level. Cu^2+^- and Au^3+^-doped systems adopt a degenerate-semiconductor profile, with a metal-derived band that approaches or crosses the Fermi level and drives the framework toward metallic behavior. So, in line with the band edge delocalization analysis, Cu, Au, Pd, Pt, Ir, and Ta fail to provide the band edge states needed to support efficient electron–hole transitions, rendering them unsuitable even when their band gaps and band alignments text appear acceptable.

### Optical Properties

Having identified the compounds with suitable band edge states, we next examined their excited response to light. In order to characterize the nature of electronic excitations, we computed the absorption spectra using the Bethe-Salpeter equation (BSE) for structures with a proper GW band gap to delve into the nature of the excitation. We characterized the exciton by analyzing the imaginary part of the dielectric function. We list the quasiparticle gap (QPG), optical gap (OPG), and exciton binding energy (EBE) from our GW-BSE calculations in Table 2 and S5. The QPG, obtained from the GW calculations, refers to the energy difference between the CB minimum (CBM) and the VB maximum (VBM), reflecting the energy required to add or remove an electron. The OPG corresponds to the lowest energy needed to create an electron–hole pair (exciton) upon light absorption and includes the effect of the Coulomb attraction between the excited electron and the hole. The EBE, defined as the difference between QPG and OPG, reflects the strength of the exciton binding. Lower EBE values indicate that excitons can be more easily dissociated into free charge carriers, which can migrate to the surface and drive reactions. Most *M*-PHI structures, except for Cu­(I), Pd, Pt, Be, and Ir, exhibit reduced EBEs compared to pristine PHI (1.1 eV), suggesting enhanced electron–hole separation in *M*-PHI, as presented in Table S5. These compounds, shown in Figure S8, exhibit nearly flat bands at the VB maximum, corresponding to a large carrier effective mass (m*), with dispersions markedly smaller than those of other *M*-PHIs. According to the simple hydrogenic (Wannier-Mott) model for exciton binding in semiconductors, the EBE scales proportionally with the effective mass:^51^

2 $$E_{bind}=\frac{m_{r}R_{H}}{\epsilon_{\infty }^{2}}$$

Where m_*r*_ is the reduced m* of the electron–hole, R_*H*_ is the Rydberg constant, and ϵ_∞_ is the dielectric constant. The flat band at the VB can stem from variations in the spatial distribution of the VB states, consistent with previous studies.^25^

**Table 2:** Quasiparticle Gap (QPG), Optical Gap (OPG), Exciton Binding Energy (EBE) (All in eV), the Ratio of Average Effective Electron m_e_* and Hole m_h_* Masses (D), and Carrier Mobility (*μ* in cm^2^/(V S)) Based on the Acoustic Deformation Potential at 300 K

	QPG	OPG	EBE	*D*	μ_*e*_	μ_*h*_
Na-PHI	3.52	2.92	0.60	12.38	3126.5	235.9
Mg-PHI	3.52	2.76	0.76	3.00	3847.4	78.06
Zn-PHI	3.46	2.75	0.71	4.02	8929	64.7
K-PHI	3.16	2.68	0.48	25.77	1669.5	522.9
Ca-PHI	3.61	2.79	0.82	6.38	3592.2	33.60
Li-PHI	3.61	2.96	0.65	3.36	5315.6	174.7

Figures 5 and S10 reveal the main trends when comparing the optical response of the M-PHI systems relative to the reference H-PHI. The latter absorbs little visible light, and Cu-PHI, in agreement with both electronic-structure calculations and experiments, performs worst of all. Most other metal dopants improve absorption by narrowing the band gap and shifting the edge into the visible range, producing a stronger optical response than H-PHI. Absorption alone, however, does not determine photocatalytic efficiency: some dopants, such as Be, achieve favorable optical gaps, but retain very high exciton binding energies (Table S5), which hinder charge separation and limit practical utility despite good absorption.

**Figure 5:**
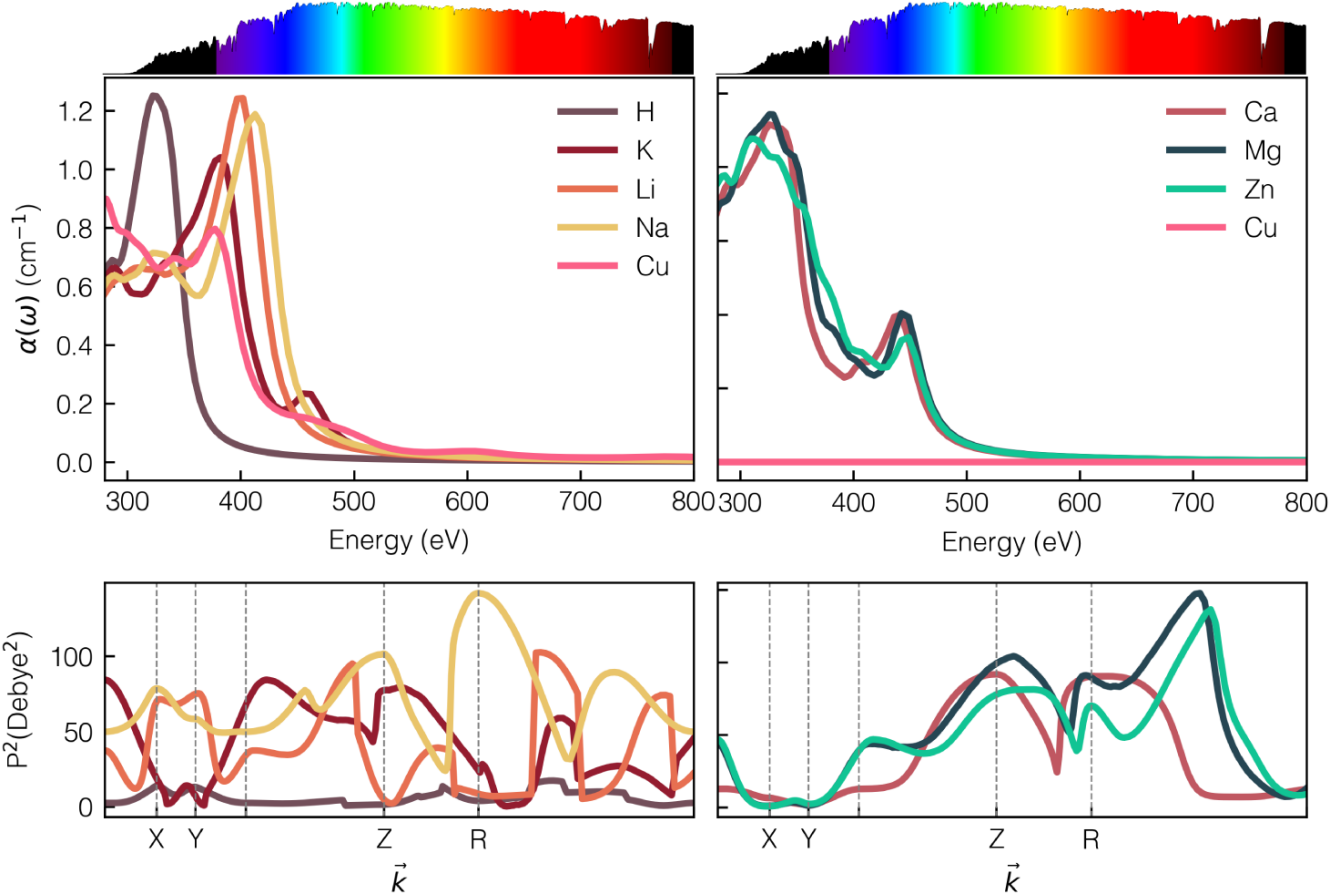
Absorption coefficient calculated by the GW/BSE method (upper panel) and transition matrix elements for selected cations (lower panel). (Right: cations 2+, left: cations 1+).

### Experimental Validation

To validate our theoretical predictions, we synthesized PHI embedded with K, Li, Na, Ca, Mg, Zn, and Cu. These metals were chosen for their abundant, cost-effective, and environmentally benign precursors, making them suitable for scalable and sustainable synthesis. In addition, their high solubility in aqueous media and well-defined coordination chemistry facilitate efficient ion exchange under mild conditions. The structures selected exhibit negative enthalpies of formation (Tables S2–S4), confirming their thermodynamic stability. Here, the GW-calculated band edges show semiquantitative consistency with experimental trends in H_2_O_2_ production, whereas both PBE and SCAN (Table S5) fail to describe these correlations. The DFT, for instance, predicts that K and Na are ineffective for H_2_O_2_ generation, while GW calculations identify them as viable photocatalysts—an observation supported by our experimental activity measurements (see Figure 6).

**Figure 6:**
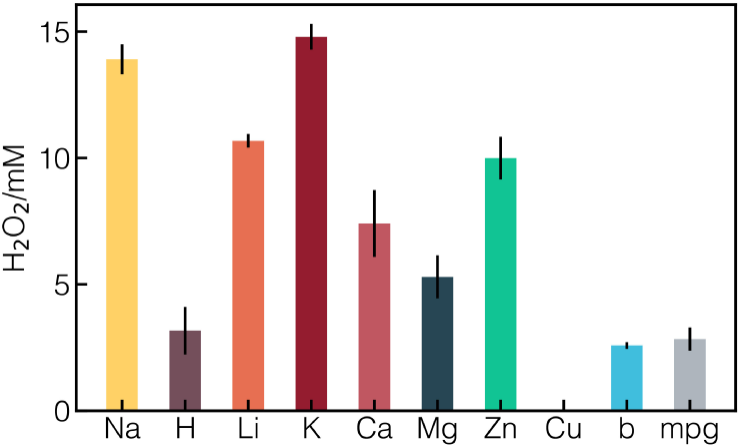
Experimental validation of some *M*-PHI and bulk g-C_3_N_4_ (b), mesoporous g-C_3_N_4_ (mpg) for H_2_O_2_ production.

We just focus on the validated M-PHI structures in the following to engender more discussion on their excitation probability and carrier mobility.

### Excitation Probability and Carrier Mobility

As predicted from the projected band structure, the structures with dominant VB of N­(p) and CB of C­(p) exhibit pronounced absorption due to their high excitation probabilities, characterized by the squared dipole transition matrix elements (*P* ^2^). The *P* ^2^ calculated at various *k*-points, also shown in Figure 5 (bottom), further support the strong transitions between the VBM and CBM of *M*-PHI.

These findings underscore the central role of exciton dynamics, an aspect that is best understood through a detailed analysis of the carrier mobility properties.

Beyond optical absorption, efficient carrier mobility plays a crucial role in enhancing photocatalytic performance. We estimated the carrier mobility by solving the linearized Boltzmann transport equation under the relaxation time approximation.^52^ Table 2 summarizes the direction-averaged acoustic deformation potentials at 300 K. The corresponding plots for different potentials in three directions are visualized in Figures S11 and S12, respectively. The mobilities in the x- and *y*-directions, where the lattice constants are nearly equal, are comparable. By contrast, the *z*-direction shows significantly lower mobility, reflecting the anisotropic nature of the crystal structure. This directional dependence confirms the anisotropy in the transport properties of the carrier. The electron mobilities calculated reach the order of 10^3^cm^2^/(V·s), surpassing those of well-known inorganic semiconductors, such as MoS_2_ (200 cm^2^/(V·s))^53^ and black phosphorus (700 cm^2^/(V·s)).^54^ Among the *M*-PHIs, Zn-PHI shows the highest electron mobility, while K-PHI exhibits the greatest hole mobility.

Carrier mobility is inherently governed by the effective mass of the charge carriers. The effective mass determines how easily electrons and holes respond to external forces, directly governing their transport properties. Our results show that electrons possess lower effective masses $\left(m_{e}^{*}\right)$ than holes, indicating faster electron transport and enabling selective charge injection in photocatalysis. We observed heavier hole effective masses and reduced mobility, linked to cell expansion and a flat VB, in systems with larger cations, such as K. The recombination rate, quantified by the ratio $D=m_{h}^{*}/m_{e}^{*}$ , reflects the balance between electron and hole dynamics.^55^ Higher *D* values imply slower recombination and, thus, better photocatalytic performance. K-PHI and Na-PHI exhibit the highest *D* values among the systems studied, suggesting superior charge separation. Although K-PHI shows a lower optical absorption, its high *D* may account for its strong H_2_O_2_ activity that we measured in experiments (see Figure 6). These findings point to a promising design strategy: combining ions to benefit from the advantages of both K-PHI and Na-PHI.

The experimental validation of H_2_O_2_ production aligned closely with our GW predictions, confirming that cation incorporation enhances the photocatalytic performance over pristine H-PHI. Alkali metals (Li, Na, K), alkaline earth metals (Mg, Ca), and Zn demonstrated the strongest activity, consistent with their favorable electronic structures, strong light absorption, and efficient carrier transport. By contrast, the inactivity of Cu-PHI reflects its poor band alignment and weak absorption, as revealed by our electronic and optical analyses. Finally, we propose the list of suitable cations for three reactions of OER, HER, ORR, and CO_2_ reduction through our GW electronic structure and optical properties in Table 3. These compounds are proposed for their band gaps exceeding 1.25 eV, their band alignments straddling the redox and oxidation potentials, and their states near the band edges being suitable, showing no intra-atomic d–d transitions, and the EBE lower than H-PHI.

**Table 3:** List of Suitable Cations for CO_2_ Reduction Reaction (CO_2_RR), Hydrogen and Oxygen Evolution Reactions (HER and OER), and the Oxygen Reduction Reaction (ORR), Respectively

M	CO_2_ RR	HER	OER	ORR
1+	Cs, K, Rb, Li, Ag	Ag, Hg, H, Li, Na, K	H, Li, Na	Li, K, Na
2+	Ba, Ca, Cd, Hg, Mg, Pb, Sr, Zn	Ba, Ca, Cd, Hg, Mg, Pb, Sn, Sr, Zn	Ca, Cd, Hg, Mg, Pb, Sn, Sr, Zn	Ba, Ca, Cd, Hg, Mg, Pb, Sn, Sr, Zn
3+	La, Sc, Y	Al, Bi, La, Sb, Sc, Y	Al, Bi, Ga, In, La, Sb, Sc, Y	Al, Bi, Ga, In, La, Sb, Sc, Y

## Conclusions

This work presents a computational framework for guiding the *in silico* engineering of PHI-based photocatalysts by the controlled incorporation of metal cations. We elucidate how the size and charge distribution of the cation influence the structural framework, modulate the electronic landscape, and improve charge mobility. Many-body perturbation theory calculations within the GW approximation were performed across a broad set of metal cations compatible with the PHI matrix. The resulting band alignments and exciton binding energies reveal structure–property relationships that underpin the photocatalytic performance.

Among the candidates screened, alkali (Li, Na, K), alkaline earth (Mg, Ca, Sr), transition (Zn, Cd), and post-transition (Sn, Pb, Bi) metals emerged as promising dopants for efficient H_2_O_2_ production. Experimental validation confirms the superior photocatalytic activity of selected systems, with Zn-, Na-, and K-incorporated PHI materials exhibiting significantly enhanced performance. This convergence of theory and experiment underscores the predictive power of the GW method and highlights the potential of rational ion selection to tune photocatalytic function. Together, our findings chart a path toward the targeted design of efficient, metal-modified PHI photocatalysts for sustainable reactions.

Looking forward, future research could explore more complex and dynamic doping strategies—such as codoping, nonmetal incorporation, or postsynthetic ion exchange—to further tailor PHI’s electronic structure and charge transport characteristics. Furthermore, the design principles established here can be extended to a wider range of solar-driven chemical transformations, including pollutant degradation. Moreover, combining theoretical screening with machine learning models could accelerate the discovery of next-generation photocatalysts with improved performance and selectivity. These directions hold great promise for advancing PHI-based materials as versatile and sustainable platforms for next-generation photocatalytic technologies.

## Methods

### Computational Methods

DFT calculations were carried out for approximately 60 screened *M*-PHI structures using the plane-wave pseudopotential method and utilizing the gradient approximation of Perdew–Burke–Ernzerhof (PBE),^56^ as implemented in the Vienna Ab initio Simulation Package (VASP).^57,58^ Projector augmented wave (PAW) pseudopotentials^59^ were used for all of our calculations. We set the cutoff energy to 520 eV to ensure the convergence of total energy and nuclear forces. All structures were fully relaxed until the force components of each atom fell below 1 meV/atom. The Brillouin zone was sampled using a Monkhorst–Pack grid density of 4 × 4 × 11 to ensure the convergence of the total energy and forces. We used the semiempirical D3 correction scheme of Grimme^60^ to account for the van der Waals interactions of layers and metal cations. In order to evaluate the thermodynamic stability, we performed ab initio molecular dynamics simulations, employing the canonical (NVT) ensemble and controlling the temperature with the Nosé-Hoover thermostat. After a 50 ps heating up to 400 K, with a sampling interval of 1 fs, the energy fluctuations were quite small (≈ ± 0.5 eV).

It is well-known that semilocal exchange-correlation functionals, such as PBE, usually underestimate the band gaps of semiconductors. Since the band gap is crucial for optical absorption and band alignment, we used the strongly constrained and appropriately normed (SCAN) meta-generalized gradient approximation (meta-GGA) method to calculate the band gaps,^61^ which, nevertheless, still has limited accuracy. Self-consistent GW (evGW) calculations were performed with DFT-GGA wave functions and eigenvalues to take into account the electronic excitations and quasiparticle (QP) energy. The dielectric constants were then calculated using the Bethe-Salpeter equation (BSE) on top of GW0 under the Tamm-Dancoff approximation^62–64^ to find the exciton binding and optical absorption coefficient. We used 600 bands and about 530 virtual states in our GW calculations, which is sufficient to converge the QP energy (the convergence plot is shown in Figure S7). The energy cutoff and the convergence threshold for electronic iterations were set to 270 eV and 10^–8^, respectively, for both GW0 and BSE calculations. We included 83 valence bands and 10 conduction bands for the BSE step.

We performed bond analyzes, such as COHP and COBI,^35^ using the LOBSTER code.^65^ Their integration up to the Fermi level (i.e., ICOHP and ICOBI) provides valuable information about the bond strength and nature of chemical bonding, respectively. The transferred charges were also calculated using the Bader method.^66^ The carrier mobility was determined by considering all electron scattering mechanisms using the AMSET package.^67^

### Experimental Details

#### Synthesis of Na-PHI

Sodium poly­(heptazine imide) (Na-PHI) was synthesized according to a procedure reported previously.^23^ In brief, 1 g of melamine was mixed with 10 g of NaCl and ground for 5 min (min) at 25 Hz using a steel ball mill vessel. The resulting mixture was transferred into a lidded porcelain crucible and placed in a furnace under a continuous nitrogen flow (5 L min^–1^). It was then heated to 600 ^◦^C at a rate of 2.3 ^◦^C/min, maintained at this temperature for 4 h, and subsequently allowed to cool to room temperature. Note that the actual temperature within the furnace chamber could fluctuate by as much as ± 50 ^◦^C. The solidified melt was removed from the crucible, placed in a 250 mL Erlenmeyer flask containing 250 mL of deionized water, and stirred overnight at room temperature. The product was then washed with approximately 1 L of deionized water using vacuum filtration, collected via centrifugation (13500 rpm, 10 min), and dried overnight in a vacuum oven at 60 ^◦^C.

#### Synthesis of H-PHI

A mixture of Na-PHI (100 mg) and HCl (20 mL, 1M) was stirred for 1.5 h. The resulting powder was then washed with H_2_O in the centrifuge six times until the pH was around 6–7 and dried in a vacuum oven at 60 ^◦^C overnight.

#### Synthesis of Li-PHI

Li-PHI was synthesized following the methodology outlined in a previous publication.^68^ We prepared a 1 M LiCl solution by dissolving 0.847 g in 20 mL of H_2_O, then gradually added a 0.1 M LiOH solution dropwise until the pH reached 9–10. Next, 0.2 g of Na-PHI was introduced into the basic solution, and the mixture was stirred at room temperature overnight. The catalyst was then washed five times with water using a centrifuge until the pH was around 6–7 and dried in a vacuum oven at 60 °C overnight.

#### Synthesis of Ca-PHI

We used the same procedure as Li-PHI for the preparation of Ca-PHI but used Ca­(OH)_2_ as a base.

#### Synthesis of K-PHI

We used the same procedure as Li-PHI for the preparation of K-PHI but used KOH as a base.

#### Synthesis of Cu-PHI, Zn-PHI, and Mg-PHI

Cu-PHI, Zn-PHI, and Mg-PHI catalysts were synthesized by taking the following part of the methodology outlined in a previous publication.^26^ We prepared a 0.1 M MCl_2_ solution by adding the salt in 15 mL of H_2_O and then 100 mg of Na-PHI was introduced to the solution.

#### Photocatalytic H_2_O_2_ Production

Photocatalytic H_2_O_2_ production experiments were conducted in a 4 mL vial reactor where, initially, 5 mg of the catalyst was dispersed in 2 mL of a 10%w/w glycerin aqueous solution, then O_2_ gas was bubbled through the mixture for 1 min. The solution was then irradiated, continuously stirring, using two purple LEDs (50 W each, λ = 410 nm) placed at a distance of 12 cm each from the reactor, for 1 h at room temperature. The resulting suspension was then centrifuged at 13300 rpm for 10 min to separate the catalyst from the solution. The amount of H_2_O_2_ generated was quantified spectrophotometrically following the titanium oxalate method.^69^ Basically, a 10 g L^–1^ solution of K_2_[TiO­(C_2_O_4_)_2_]·2H_2_O was prepared using 450 mL of water and 50 mL of sulfuric acid to avoid complex precipitation. A amount of 1.5 mL of this complex was then mixed with 0.5 mL of the supernatant from the photocatalytic reaction. The prepared solutions were analyzed with a UV–vis spectrometer, after appropriate dilution when necessary, measuring the absorbance at 400 nm. In order to ensure accuracy, a calibration curve was constructed using external samples with known H_2_O_2_ concentrations ranging from 0 to 10 mmol L^–1^, yielding a highly linear analytical response (*R* ^2^ = 0.99996). At least three independent replicates per sample for the H_2_O_2_ generation data were done which improved the statistical confidence and reproducibility of our findings.

## Supplementary Material



## Notes

The list of materials studied, along with their structural properties, electronic properties, band structure, density of states, and optical absorption plots (PDF)
