# Supplementary material for: Theory-Guided Discovery of Ion-Exchanged Poly(heptazine imide) Photocatalysts Using First-Principles Many-Body Perturbation Theory
Source: J Am Chem Soc. 2026 Jan 7;148(2):2165–74. doi: 10.1021/jacs.5c09930 (PMC12833806; doi:10.1021/jacs.5c09930)
Supplement: Supplementary file 1 [file ja5c09930_si_001.pdf]

Theory-Guided Discovery of Ion-Exchanged Poly(heptazine Imide) Photocatalysts Using First-Principles Many-Body Perturbation Theory

Zahra Hajiahmadi*[1], Anna Lo Presti[2], S. Shahab Naghavi[3], Markus Antonietti[2], Christian Mark Pelicano[2], and Thomas D. Kühne[1]

[1]CASUS - Center for Advanced Systems Understanding, Helmholtz-Zentrum Dresden-Rossendorf e.V. (HZDR), Untermarkt 20, D-02826 Görlitz, Germany
[2]Department of Colloid Chemistry, Max Planck Institute of Colloids and Interfaces, D-14476 Potsdam, Germany
[3]Department of Physical and Computational Chemistry, Shahid Beheshti University, Tehran 1983969411, Iran

Table S 1: Atomic radii (r_a (Å)), Ionic radii (r_i (Å)), Electronegativity (EN (Pauling)), Lattice parameters (Å, deg), Cell volume (Å^3), and geometrical changes, void dimention (d_1, d_2) Å, Angle of distortion (δ)°

M	r_a	r_i	EN	a	b	c	α	β	γ	Volume	Distortion	d_1 , d_2	δ
Ag^+	1.65	1.15	1.93	12.55	12.56	4.54	112.17	112.03	119.74	411.08	Distorted-Interlayer	12.56, 9.92	169.85
Au^+	1.74	1.37	2.54	12.48	12.48	4.50	111.79	111.64	120.25	405.46	Distorted-Interlayer	12.48, 9.74	168.02
Cs^+	2.98	1.67	0.79	13.17	13.17	5.75	114.34	119.93	119.93	489.67	No Distortion-In plane	13.17, 10.66	176.81
Cu^+	1.45	0.77	1.9	12.24	12.25	4.72	112.95	112.63	118.83	402.47	Distorted-Interlayer	12.25, 9.10	166.65
H^+	0.53		2.2	12.94	12.94	4.67	112.70	111.60	120.00	456.65		12.94, 10.28	177.58
Hg^+	1.71	1.19	2.0	12.79	12.79	4.37	111.11	112.28	119.55	422.21	Distorted-Interlayer	12.79, 10.25	173.83
K^+	2.43	1.38	0.82	13.06	13.06	5.81	114.79	115.03	119.68	469.63	No Distortion-In plane	13.06, 10.55	178.14
Li^+	1.67	0.76	0.98	12.60	12.60	4.73	112.67	112.65	119.24	424.63	Distorted-Interlayer	12.60, 10.67	169.00
Na^+	1.90	1.02	0.93	12.65	12.56	4.35	108.42	113.35	119.50	405.46	No Distortion-In plane	12.56, 10.06	175.13
Rb^+	2.65	1.52	0.82	13.16	13.16	5.98	115.03	114.93	119.85	483.54	No Distortion-In plane	13.16, 10.65	177.50
Ag^{+2}	1.65	0.94	1.93	12.70	12.83	4.94	132.49	92.36	120.05	413.68	No Distortion-Interlayer	12.83, 9.96	174.5
Ba^{+2}	2.53	1.35	0.89	12.38	12.68	5.19	139.64	86.79	120.25	405.46	Distorted-In plane	12.68, 9.83	167.81
Be^{+2}	1.12	0.45	1.57	12.64	12.45	4.55	93.33	119.07	119.93	489.67	Distorted-Interlayer	12.45, 9.97	167.44
Ca^{+2}	1.94	1.0	1.0	12.75	12.74	4.57	115.775	107.35	118.83	402.47	Distorted-In plane	12.74, 10.09	173.8
Cd^{+2}	1.61	0.95	1.69	12.69	12.86	4.88	132.97	91.62	119.89	411.57	No Distortion-Interlayer	12.86, 9.97	176.15
Co^{+2}	1.52	0.65	1.88	12.67	12.82	4.64	122.70	101.57	120.35	415.17	No Distortion-Interlayer	12.82, 9.31	177.34
Cr^{+2}	1.66	0.73	1.66	12.74	12.86	4.53	119.96	103.25	120.71	420.26	No Distortion- Interlayer	12.86, 9.97	178.40
Cu^{+2}	1.45	0.73	1.90	12.70	12.83	5.00	128.17	97.77	120.40	415.07	No Distortion-Interlayer	12.84, 9.89	176.15
Fe^{+2}	1.56	0.61	1.83	12.72	12.84	4.51	121.73	101.64	120.68	413.39	No Distortion- Interlayer	12.84, 9.04	178.10
Ge^{+2}	1.25	0.73	2.01	12.69	12.77	5.51	128.02	101.20	119.91	413.88	Distorted-Interlayer	12.77, 9.99	168.83
Hg^{+2}	1.71	1.02	2.0	12.55	12.80	5.10	137.56	87.55	119.93	397.85	No Distortion-In plane	12.80, 9.82	176.35
Mg^{+2}	1.45	0.72	1.31	12.71	12.86	4.72	128.52	95.51	120.00	415.65	Distorted-Interlayer	12.86, 9.98	173.80
Mn^{+2}	1.61	0.83	1.55	12.73	12.75	4.53	120.61	102.66	120.72	418.019	No Distortion-Interlayer	12.85, 9.90	178.40
Ni^{+2}	1.49	0.69	1.91	12.61	12.80	4.92	128.09	97.52	120.34	408.90	No Distortion-Interlayer	12.80, 9.79	176.40
Pd^{+2}	1.69	0.86	2.20	12.55	12.58	5.06	127.75	97.61	120.49	413.81	No Distortion- In plane	12.58, 9.79	175.51
Pt^{+2}	1.77	0.80	2.28	12.57	12.55	4.96	124.65	99.63	120.73	421.58	No Distortion- In plane	12.55, 9.66	174.47
Sn^{+2}	1.45	1.18	1.96	12.73	12.83	5.04	129.49	96.74	120.02	417.34	No Distortion- Interlayer	12.83, 10.02	175.85
Sr^{+2}	2.19	1.18	0.95	12.36	12.63	5.42	138.25	90.12	119.22	382.27	Distorted-In plane	12.63, 9.76	167.38
V^{+2}	1.71	0.79	1.63	12.76	12.85	4.52	1114.92	108.24	121.03	426.68	No Distortion-Interlayer	12.85, 7.78	178.71
Zn^{+2}	1.42	0.74	1.65	12.68	12.84	4.84	129.54	95.40	120.37	409.48	No Distortion-Interlayer	12.84, 9.87	175.29
Pb^{+2}	1.54	1.19	2.33	12.74	12.43	5.20	87.42	139.40	119.12	384.60	Distorted- In plane	12.79, 9.98,	162.72
Al^{+3}	1.18	0.53	1.61	12.26	13.06	5.94	123.83	104.67	122.43	411.79	Distorted- In plane	11.90, 9.59	171.48
Au^{+3}	1.74	0.84	2.54	12.76	12.69	4.67	117.10	105.84	120.54	436.91	No Distortion-In plane	12.69, 9.97	177.02
Bi^{+3}	1.43	1.03	2.02	12.53	12.65	5.20	121.13	102.48	121.31	451.26	Distorted-In plane	12.65, 9.68	167.99
Co^{+3}	1.52	0.55	1.88	12.57	12.66	4.48	119.76	103.07	120.20	410.16	No Distortion-Interlayer	12.66, 10.82	175.29
Cr^{+3}	1.66	0.62	1.66	12.55	12.65	4.45	117.96	104.13	120.57	415.63	No Distortion-Interlayer	12.65, 9.75	178.15
Fe^{+3}	2.98	0.55	1.83	12.58	12.69	4.43	119.97	102.13	120.84	409.95	No Distortion-Interlayer	12.69, 9.74	177.76
Ga^{+3}	1.36	0.62	1.81	12.61	12.49	4.69	114.51	107.33	120.27	444.05	Distorted- In plane	12.49, 9.88	171.60
In^{+3}	1.56	0.80	1.78	12.43	12.41	4.62	109.68	113.63	118.76	430.34	Distorted-In plane	12.41, 9.90	172.3
Ir^{+3}	1.80	0.68	2.20	12.71	12.68	5.65	124.34	105.02	120.34	419.49	Distorted-Interlayer	12.68, 10.10	167.19
La^{+3}	1.95	1.06	1.10	12.25	11.63	4.33	95.53	123.33	117.02	406.54	No Distortion-In plane	11.63, 10.16	176.15
Mn^{+3}	2.98	0.58	1.55	12.56	12.68	4.43	118.92	102.46	121.05	415.50	No Distortion-Interlayer	12.68, 9.71	176.16
Mo^{+3}	1.90	0.69	2.16	12.17	12.03	4.57	104.34	117.21	118.11	423.47	Distorted-In plane	12.03, 9.74	174.44
Nb^{+3}	1.98	0.69	1.60	12.38	10.79	4.63	90.56	129.90	115.58	389.93	Distorted-In plane	10.79, 10.79	164.9
Ni^{+3}	1.49	0.69	1.91	12.40	12.66	4.83	129.91	94.88	120.06	398.25	No Distortion-Interlayer	12.66, 9.56	175.02
Pd^{+3}	1.69	0.76	2.20	12.23	12.35	4.81	126.79	97.70	119.63	397.79	No Distortion-In-plane	12.35, 9.36	175.78
Rh^{+3}	1.73	0.67	2.28	12.68	12.76	4.40	119.95	101.79	120.53	420.13	No Distortion-Interlayer	12.76, 9.88	179.01
Ru^{+3}	1.78	0.68	2.20	12.66	12.78	4.36	120.36	100.77	120.75	418..86	No Distortion-Interlayer	12.78, 9.83	179.18
Sb^{+3}	1.33	0.76	2.05	12.72	12.77	4.49	118.47	104.19	119.73	432.09	Distorted-In plane	12.77, 10.09	172.90
Sc^{+3}	1.84	0.75	1.36	12.46	11.17	4.51	90.59	129.67	116.42	391.33	Distorted-In plane	11.17, 10,65	165.87
Ta^{+3}	2.00	0.72	1.50	11.66	11.16	4.77	100.44	126.30	113.35	380.33	Distorted-In plane	11.16, 9.62	164.04
Ti^{+3}	1.76	0.67	1.54	12.32	10.90	4.54	91.85	129.99	115.49	377.48	Distorted-In plane	10.90, 10.67	163.89
Tl^{+3}	1.56	1.5	1.62	12.42	12.19	4.88	119.49	107.48	118.94	398.05	Distorted-Interlayer	12.19,9.94	167.79
V^{+3}	1.71	0.64	1.63	12.10	11.97	4.60	105.64	116.73	118.47	412.38	Distorted-In plane	11.97, 9.61	173.33
Y^{+3}	2.12	0.9	1.22	12.27	11.43	4.34	94.59	125.35	116.47	393.86	Distorted-In plane	11.43, 10.32	163.45

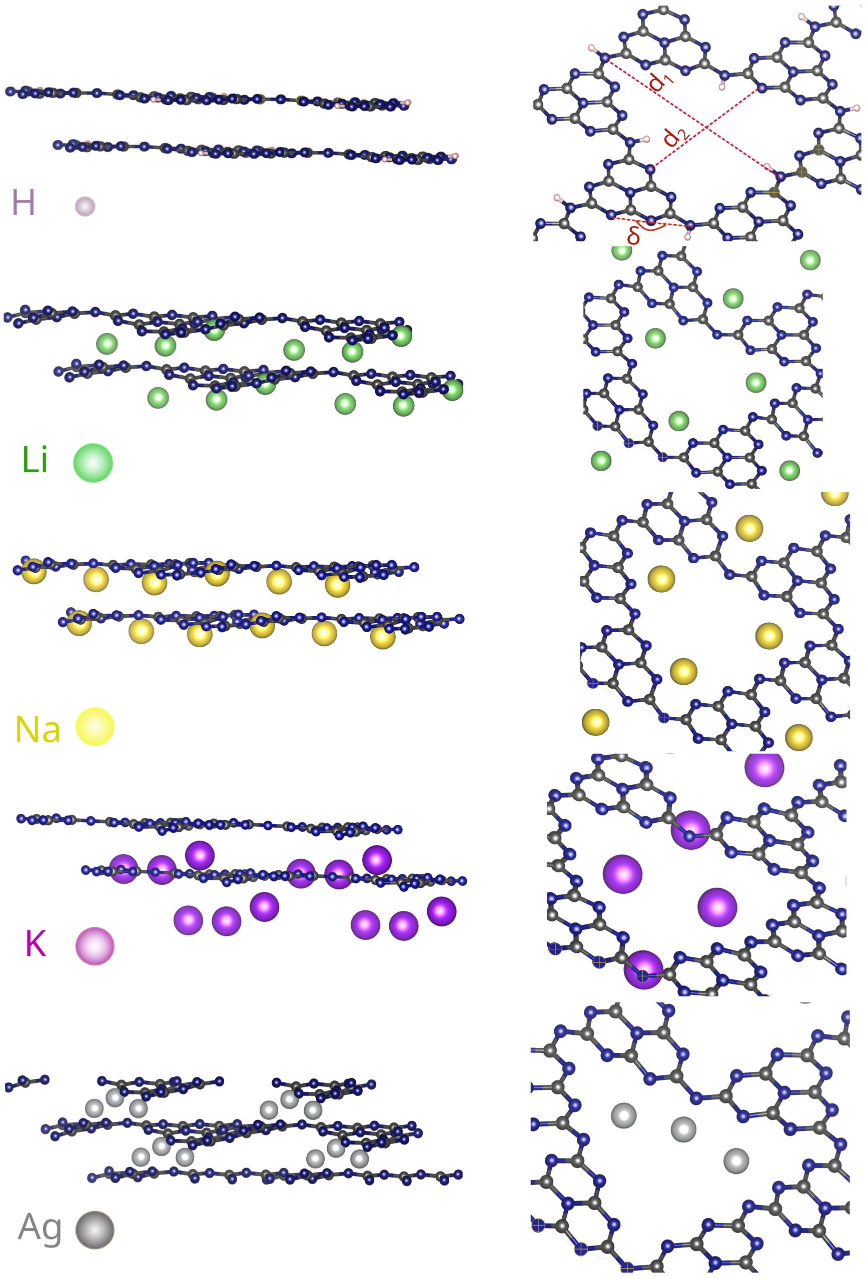

Fig. S1: Side view and top view of some M-PHI (M=cation +1)

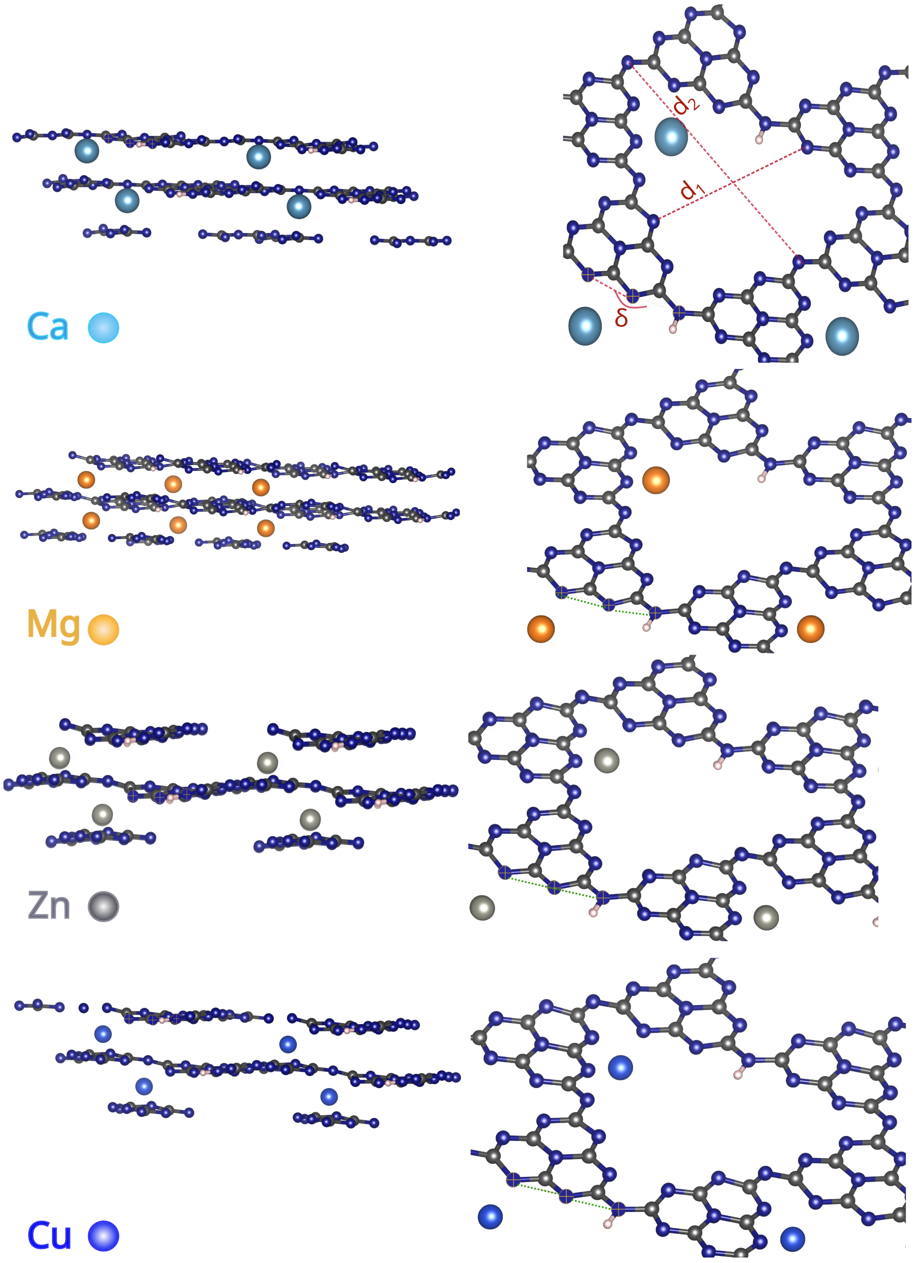

Fig. S2: Side view and top view of some M-PHI (M=cation +2)

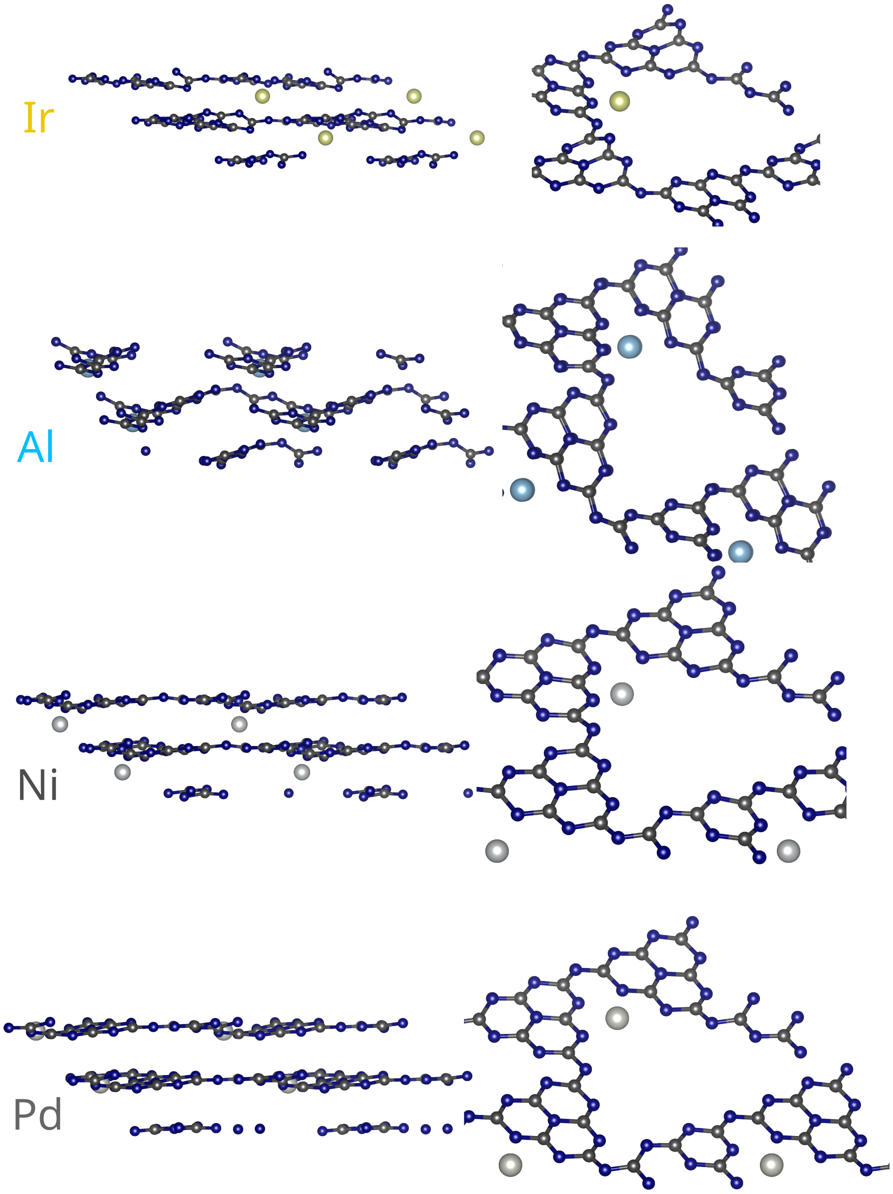

Fig. S3: Side view and top view of some M-PHI (M=cation +3)

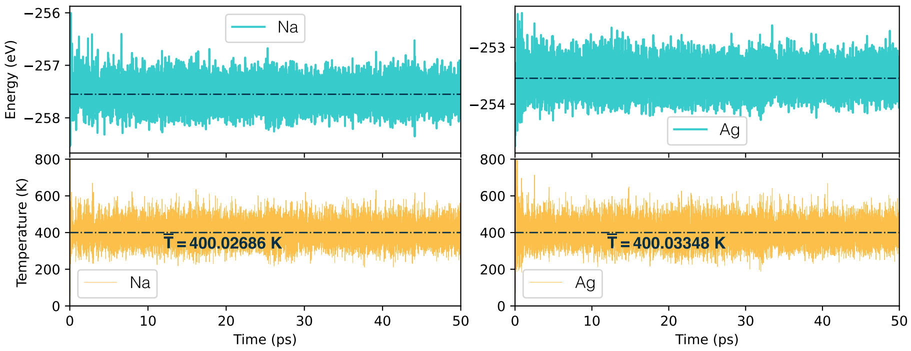

Fig. S 4: Variations of temperature and energy in the AIMD simulations of the Na-PHI and Ag-PHI at 400 K

$$\mathrm{COHP}_{AB}(E) = \sum_{n,\mathbf{k}} f_{n\mathbf{k}}\, c^*_{n\mathbf{k},A}\, H_{AB}(\mathbf{k})\, c_{n\mathbf{k},B}\; \delta(E - \epsilon_{n\mathbf{k}}) \tag{1}$$

$$\mathrm{ICOHP}_{AB} = \int_{-\infty}^{E_F} \mathrm{COHP}_{AB}(E)\, dE \tag{2}$$

$$\mathrm{COBI}_{AB}(E) = \sum_{n,\mathbf{k}} f_{n\mathbf{k}}\, c^*_{n\mathbf{k},A}\, S_{AB}(\mathbf{k})\, c_{n\mathbf{k},B}\; \delta(E - \epsilon_{n\mathbf{k}}) \tag{3}$$

$$\mathrm{ICOBI}_{AB} = \int_{-\infty}^{E_F} \mathrm{COBI}_{AB}(E)\, dE \tag{4}$$

n = band index k = k-point $f_{n\mathbf{k}}$ = occupation number of band n at k-point k $c_{n\mathbf{k}}$= coefficient of orbital on atom A in Bloch function ψ_{nk} $H_{AB}(\mathbf{k})$ = Hamiltonian matrix element between orbitals on atoms A and B $\epsilon_{n\mathbf{k}}$ = eigenvalue (band energy) δ = Dirac delta (energy resolution) $H_{AB}(\mathbf{k})$ = the overlap matrix element between orbitals on A and B

$$\Delta H_f = E(M\text{-}PHI)+2nE(H_2)\text{-}E(PHI)\text{-}nE(M_{atom}) \tag{5}$$

Table S 2: -ICOHP (eV), ICOBI (eV), and Bader C-N, N-M, and C-M (M=Cation +1) bonds, Enthalpy of formation energy (ΔH_f) (eV)

M^+	Bond Analysis	C-N	N-M	C-M	M-M	ΔH_f
	−ICOHP	13.31	1.57	0.08	0.57	
Ag^+	ICOBI	1.26	0.27	0.009	0.16	-1.01
	Bader	N:6.17	M:10.46		CDD M: 1.61/3	
	−ICOHP	13.12	2.40	0.11		
Au^+	ICOBI	1.23	0.35	0.14	0.28	-0.61
	Bader	N:6.13	M:10.60		CDD M: 1.20/3	
	−ICOHP	9.87	0.129	0.0101	0.295	
Cs^+	ICOBI	1.11	0.023	0.0076	0.034	-5.37
	Bader	N:6.28	M:8.15		CDD M: 2.55/3	
	−ICOHP	13.21	1.69	0.86	0.639	
Cu^+	ICOBI	1.26	0.32	0.014	0.213	-3.56
	Bader	N:0.30	M:10.43		CDD M: 1.71/3	
	−ICOHP	12.36	0.05	0.080		
H^+	ICOBI	1.14	0.82	0.002		
	Bader	N:6.22	M:0.490			
	−ICOHP	13.08	1.096	0.052	1.24	
Hg^+	ICOBI	1.19	0.17	0.0055	0.299	1.97
	Bader	N:6.14	M:11.50		CDD M: 1.50/3	
	−ICOHP	12.44	0.27	0.076	0.003	
K^+	ICOBI	1.29	0.055	0.006	0.0018	-4.87
	Bader	N:2.48	M:6.14		CDD M: 2.58/3	
	−ICOHP	13.14	0.55	0.15	0.01	
Li^+	ICOBI	1.30	0.08	0.010	0.0005	-8.57
	Bader	N:6.25	M:0.12		CDD M:2.64/3	
	−ICOHP	13.57	0.38	0.09	0.003	
Na^+	ICOBI	1.30	0.08	0.007	0.002	-6.55
	Bader	N:6.23	M:0.145		CDD M: 2.56/3	
	−ICOHP	13.08	0.23	0.059	0.05	
Rb^+	ICOBI	1.26	0.04	0.007	0.006	-4.77
	Bader	N:6.27	M:6.17		CDD M: 2.58/3	

Table S3: -ICOHP (eV), ICOBI (eV), and Bader C-N, N-M, and C-M (M=Cation +2) bonds, Enthalpy of formation energy (ΔH_f) (eV)

M^{+2}	Bond Analysis	C-N	N-M	C-M	ΔH_f
	−ICOHP	12.83	0.36	0.063	
Ba^{+2}	ICOBI	1.30	0.39	0.008	-4.22
	Bader	N:6.27	M:8.36	CDD M: 1.64	
	−ICOHP	11.92	1.66	0.154	
Be^{+2}	ICOBI	1.067	0.24	0.0065	-2.58
	Bader	N:6.41	M:0.31	CDD M: 1.69	
	−ICOHP	12.89	0.425	0.091	
Ca^{+2}	ICOBI	1.34	0.075	0.0073	-3.46
	Bader	N:6.27	M:6.43	CDD M: 1.57	
	−ICOHP	13.84	1.32	0.042	
Cd^{+2}	ICOBI	1.38	0.273	0.0038	-1.92
	Bader	N:6.18	M:10.81	CDD M: 1.19	
	−ICOHP	13.82	1.81	0.042	
Cu^{+2}	ICOBI	1.34	0.26	0.0038	-0.33
	Bader	N:6.16	M:10.08	CDD M: 0.918	
	−ICOHP	13.72	2.54	0.040	
Fe^{+2}	ICOBI	1.30	0.434	0.078	-3.14
	Bader	N:6.12	M:6.93	CDD M:1.07	
	−ICOHP	14.19	1.87	0.036	
Hg^{+2}	ICOBI	1.43	0.35	0.006	2.95
	Bader	N:6.09	M:11.05	0.94	
	−ICOHP	13.69	1.05	0.95	
Mg^{+2}	ICOBI	1.34	0.18	0.0057	-5.33
	Bader	N:0.41	M:0.28	CDD M: 1.66	
	−ICOHP	13.84	2.26	0.21	
Ni^{+2}	ICOBI	1.33	0.47	0.31	-1.91
	Bader	N:0.31	M:9.096	CDD M: 0.90	
	−ICOHP	13.08	1.56	0.153	
Pb^{+2}	ICOBI	1.21	0.29	0.17	-1.90
	Bader	N:0.34	M:2.71	CDD M: 1.28	
	−ICOHP				
Pd^{+2}	ICOBI				0.28
	Bader	N:	M:9.23	CDD M: 0.77	
	−ICOHP				
Pt^{+2}	ICOBI				-1.25
	Bader	N:	M:9.33	CDD M: 0.66	
	−ICOHP	13.65	2.24		
Sn^{+2}	ICOBI	1.35	0.416		-2.08
	Bader	N:0.30	M:2.72	CDD M: 1.28	
	−ICOHP	13.35			
Sr^{+2}	ICOBI	1.33			-3.84
	Bader	N:	M:8.35	CDD M: 1.64	
	−ICOHP	13.85	1.66	0.0060	
Zn^{+2}	ICOBI	1.37	0.28	0.0053	0.50
	Bader	N: 6.23	M:10.79	CDD M:1.21	

Table S4: −ICOHP (eV), ICOBI (eV), and Bader C-N, N-M, and C-M (M=Cation +3) bonds, Enthalpy of formation energy (ΔH_f) (eV)

M^{+2}	Bond Analysis	C-N	N-M	C-M	ΔH_f
	−ICOHP	13.88	2.48	0.68	
Al^{+3}	ICOBI	1.40	0.28	0.053	0.93
	Bader	N:6.45	M:0.625	CDD M: 2.37	
	−ICOHP	14.12	2.46	0.10	
Au^{+3}	ICOBI	1.41	0.35	0.011	
	Bader	N:	M:10.201	CDD M: 0.79	
	−ICOHP	13.95	3.02	0.13	
Bi^{+3}	ICOBI	1.43	0.60	0.011	0.06
	Bader	N:6.23	M: 3.33	CDD M: 1.67	
	−ICOHP	14.38	2.51	0.25	
CO^{+3}	ICOBI	1.42	0.45	0.36	-0.60
	Bader	N:6.12	M:7.93	CDD M: 1.067	
	−ICOHP	14.42	3.13	0.41	
Cr^{+3}	ICOBI	1.45	0.68	0.06	-2.11
	Bader	N: 6.13	M: 4.58	CDD M: 1.42	
	−ICOHP	14.40	2.65	0.28	
Fe^{+3}	ICOBI	1.43	0.60	0.46	-0.89
	Bader	N:6.10	M:6.83	CDD M:1.16	
	−ICOHP	14.28	4.76	0.59	
Ga^{+3}	ICOBI	1.45	0.60	0.048	1.73
	Bader	N: 6.20	M:1.49	CDD M:1.51	
	−ICOHP	13.97	3.25	0.42	
In^{+3}	ICOBI	1.39	0.41	0.035	2.35
	Bader	N:6.18	M:1.55	CDD M: 1.45	
	−ICOHP	13.90	3.47	0.13	
Ir^{+3}	ICOBI	1.40	0.70	0.029	-1.47
	Bader	N:6.58	M: 7.93	CDD M:1.07	
	−ICOHP	12.99	2.48	0.56	
La^{+3}	ICOBI	1.38	0.42	0.05	-5.54
	Bader	N:6.25	M: 8.93	CDD M: 2.07	
	−ICOHP	14.43	3.0	0.37	
Mn^{+3}	ICOBI	1.45	0.66	0.069	-1.45
	Bader	N:6.15	M:5.71	CDD M: 1.29	
	−ICOHP	13.83	3.67	0.54	
Nb^{+3}	ICOBI	1.37	0.61	0.06	-5.92
	Bader	N:6.22	M:10.93	CDD M:2.06	
	−ICOHP	14.13	2.37	0.16	
Ni^{+3}	ICOBI	1.38	0.40	0.02	0.78
	Bader	N:6.08	M:9.026	CDD M:0.97	
	−ICOHP	14.44	2.84	0.17	
Rh^{+3}	ICOBI	1.44	0.47	0.02	0.27
	Bader	N:6.03	M:8.026	CDD M: 0.97	
	−ICOHP	14.44	3.56	0.22	
Ru^{+3}	ICOBI	1.44	0.69	0.037	-1.27
	Bader	N: 6.04	M:6.90	CDD M: 1.10	

M^{+2}	Bond Analysis	C-N	N-M	C-M	ΔH_f
	−ICOHP	14.28	3.37	0.14	
Sb^{+3}	ICOBI	1.46	0.55	0.15	0.92
	Bader	N:6.16	M:3.31	CDD M: 1.68	
	−ICOHP	13.95	2.42	0.42	
Sc^{+3}	ICOBI	1.39	0.48	0.047	-5.29
	Bader	N:6.21	M:1.22	CDD M: 1.78	
	−ICOHP	13.97	2.65	0.42	
Ti^{+3}	ICOBI	1.38	0.56	0.05	-4.93
	Bader	N:6.22	M:2.27	CDD M:1.73	
	−ICOHP	13.95	1.05	0.89	
Tl^{+3}	ICOBI	1.36	0.22	0.01	3.41
	Bader	N:6.12	M:2.24	CDD M:0.75	
	−ICOHP	14.05	2.61	0.51	
V^{+3}	ICOBI	1.40	0.52	0.078	-3.35
	Bader	N:6.14	M:3.43	CDD M:1.57	
	−ICOHP	14.04	2.46	0.23	
Y^{+3}	ICOBI	1.39	0.412	0.02	-6.81
	Bader	N: 6.26	M: 8.86	CDD M:2.14	

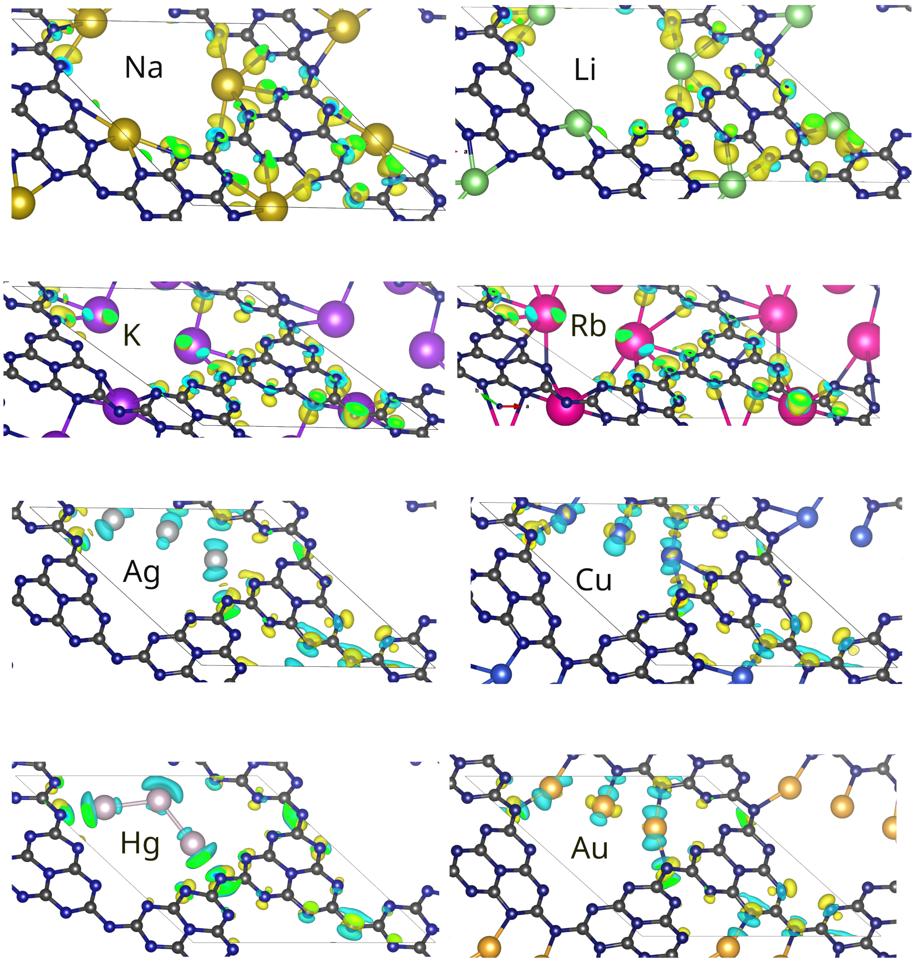

Fig. S5: Charge density differences plot for some M-PHI (M=cation +1)

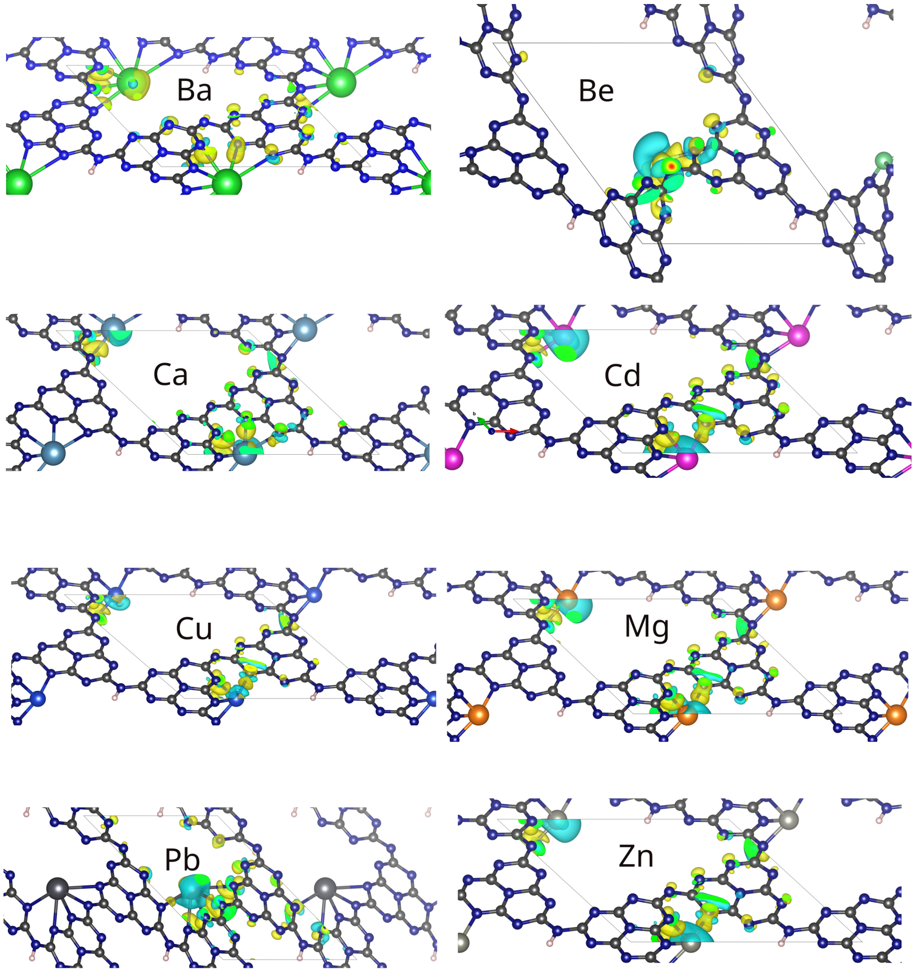

Fig. S6: Charge density differences plot for some M-PHI (M=cation +2)

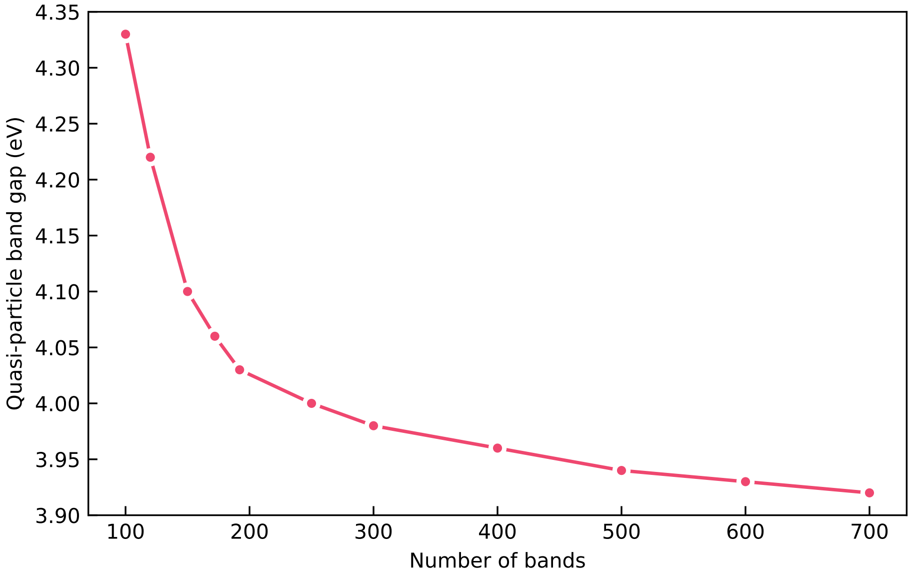

Fig. S 7: Convergence of the quasiparticle band gap of H-PHI with respect to the number of total bands

Table S 5: Band-Gap and Bandalignment with PBE, SCAN, and GW methods, Optical bandgap and Exciton binding energy (EBE) in eV

M^{+}	E_{BG}(PBE)	VB(PBE)	CB(PBE)	E_{BG}(SCAN)	VB(SCAN)	CB(SCAN)	E_{BG}(GW)	VB(GW)	CB(GW)	Optical BG	EBE
Ag^{+}	1.65	-5.058	-3.40	1.88	-5.37	-3.49	2.75	-4.92	-2.11	2.26	0.54
Au^{+}	1.11	-4.701	-3.58	0.80	-	-	2.37	-4.70	-2.21	2.09	0.39
Cs^{+}	1.80	-3.910	-2.11	2.12	-4.127	-2.01	3.05	-3.34	-0.09	2.71	0.47
Cu^{+}	1.37	-4.614	-3.25	1.53	-4.810	-3.28	2.56	-4.69	-2.05	1.42	1.22
H^{+}	2.21	-7.269	-5.06	2.56	-7.519	-4.96	3.93	-7.01	-2.86	2.94	1.11
Hg^{+}	1.65	-4.840	-3.19	2.06	-5.20	-3.14	2.14	-5.33	-3.66		
K^{+}	1.76	-4.188	-2.43	2.08	-4.40	-2.33	3.16	-5.13	-0.97	2.68	0.48
Li^{+}	2.13	-6.640	-4.50	2.49	-6.51	-4.08	3.61	-6.23	-2.45	2.96	0.65
Na^{+}	2.08	-7.443	-5.36	2.43	-7.71	-5.28	3.52	-7.06	-3.28	2.92	0.60
Rb^{+}	1.86	-3.72	-1.86	2.18	-3.95	-1.76	3.16	-3.22	0.10	2.69	0.59
Ag^{+2}	0.0	-	-	0.03	-	-	0.13	-	-	-	-
Ba^{+2}	1.84	-5.45	-3.61	2.16	-5.70	-3.53	3.18	-5.11	-1.65	2.74	0.55
Be^{+2}	2.15	-5.90	-3.75	2.53			4.13	-5.88	-1.63	2.92	1.26
Ca^{+2}	2.02	-5.77	-3.75	2.35	-5.98	-3.64	3.49	-5.42	-1.66	2.79	0.82
Cd^{+2}	2.0	-5.65	-3.65	2.32	-5.88	-3.55	3.37	-5.26	1.66	2.73	0.64
Co^{+2}	0.32	-	-	0.47	-	-	0.22	-	-	-	-
Cr^{+2}	-0.01	-	-	0.31	-	-	0.00	-	-	-	-
Cu^{+2}	0	-	-	0	-	-	0.3	-	-	-	-
Fe^{+2}	0.0	-	-	1.21	-	-	0.98	-	-	-	-
Ge^{+2}	1.86	-7.65	-5.79	2.19	-7.92	-5.73	3.21	-7.09	-6.78	2.68	0.63
Hg^{+2}	1.93	-5.86	-3.94	2.10	-6.12	-4.02	3.17	-5.56	-2.10	2.67	0.57
Mg^{+2}	2.0	-5.62	-3.62	2.32	-5.85	-3.53	3.42	-5.25	-1.61	2.76	0.76
Mn^{+2}	-0.02	-	-	0.98	-	-	0.00	-	-	-	-
Ni^{+2}	0.26	-	-	0.63	-	-	0.32	-	-	-	-
Pd^{+2}	1.02	-	-	1.31	-7.52	-6.21	2.07	-7.02	-4.51	1.28	1.23
Pt^{+2}	1.52	-7.28	-5.77	1.95	-7.59	-5.64	2.95	-7.10	-4.04	1.70	1.35
Sn^{+2}	1.83	-6.49	-4.66	2.13	-6.74	-4.60	3.14	-6.15	-2.84	2.59	0.65
Sr^{+2}	1.73	-5.76	-4.03	2.05	-6.01	-3.96	3.07	-5.59	-2.18	2.69	0.48
V^{+2}	0.0	-	-	0.09	-	-	-	-	-	-	-
Zn^{+2}	1.96	-5.56	-3.60	2.28	-5.80	-3.52	3.35	-5.27	-1.70	2.75	0.71
Pb^{+2}	1.69	-5.75	-4.06				2.93	-5.45	-2.13	2.62	0.40
Al^{+3}	1.96	-6.66	-4.70	1.98	-6.65	-4.69	2.22	-5.51	-3.07	2.07	0.37
Au^{+3}	0.02	-	-	0.08	-	-	-0.12	-	-	-	-
Bi^{+3}	1.77	-7.13	-5.36	1.75	-7.13	-5.36	3.22	-7.05	-3.74	2.43	0.88
Co^{+3}	0.01	-	-	0.06	-	-	0.99	-	-	-	-
Cr^{+3}	0.03	-	-	0.37	-	-	0	-	-	-	-
Fe^{+3}	0.29	-	-	0.0	-	-	-0.13	-	-	-	-
Ga^{+3}	1.54	-6.99	-5.66	1.54	-6.99	-5.66	2.67	-6.76	-4.09	1.98	0.69
In^{+3}	0.87	-	-	1.01	-	-	1.98	-6.24	-4.27	1.39	0.59
Ir^{+3}	0.89	-	-	1.25	-6.22	-4.96	2.0	-5.57	-3.57	0.88	1.12
La^{+3}	1.97	-5.56	-3.59	2.32	-5.81	-3.49	3.59	-5.24	-1.52	3.012	0.71
Mn^{+3}	0.03	-	-	0.13	-	-	-0.5	-	-	-	-
Mo^{+3}	0.23	-	-	0.47	-	-	-	-			
Nb^{+3}	-0.21	-	-	-0.19	-	-	0.0	-	-	-	-
Ni^{+3}	-0.01	-	-	0.75	-	-	-0.13	-	-	-	-
Pd^{+3}	0.01	-	-	0.02	-	-	-	-	-	-	-
Rh^{+3}	0.14	-	-	0.21	-	-	-0.14	-	-	-	-
Ru^{+3}	0.58	-	-	1.02	-	-	0.11	-	-	-	-
Sb^{+3}	0.13	-	-	1.46	-6.67	-5.21	2.38	-6.11	-3.73	1.82	0.56
Sc^{+3}	2.17	-6.58	-4.41	2.53	-6.85	-4.32	3.81	-6.28	-2.47	2.93	0.88
Ta^{+3}	0.89	-	-	1.11	-	-	1.77	-5.79	-4.00	1.21	0.56
Ti^{+3}	-0.12	-	-	-0.07	-	-	0	-	-	-	-
Tl^{+3}	-0.07	-	-	-0.08	-	-	0.0	-	-	-	-
V^{+3}	-0.02	-	-	-0.02	-	-	1.54	-5.84	-4.29	0.35	1.19
Y^{+3}	2.12	-5.78	-3.66	2.48	-6.05	-3.57	3.84	-5.53	-1.69	2.93	0.91

Table S6: Band-Gap and Bandalignment for distortion effect

M^+	E_{BG}(PBE)	VB(PBE)	CB(PBE)
Na^+	2.08	-7.44	-3.36
Rb^+	1.86	-3.72	-1.87
Na^+ in Rb^+ structure	1.82	-3.57	-1.75
Au^+	1.11	-4.70	-3.58
Na^+ in Au^+ structure	1.3	-4.55	-3.25

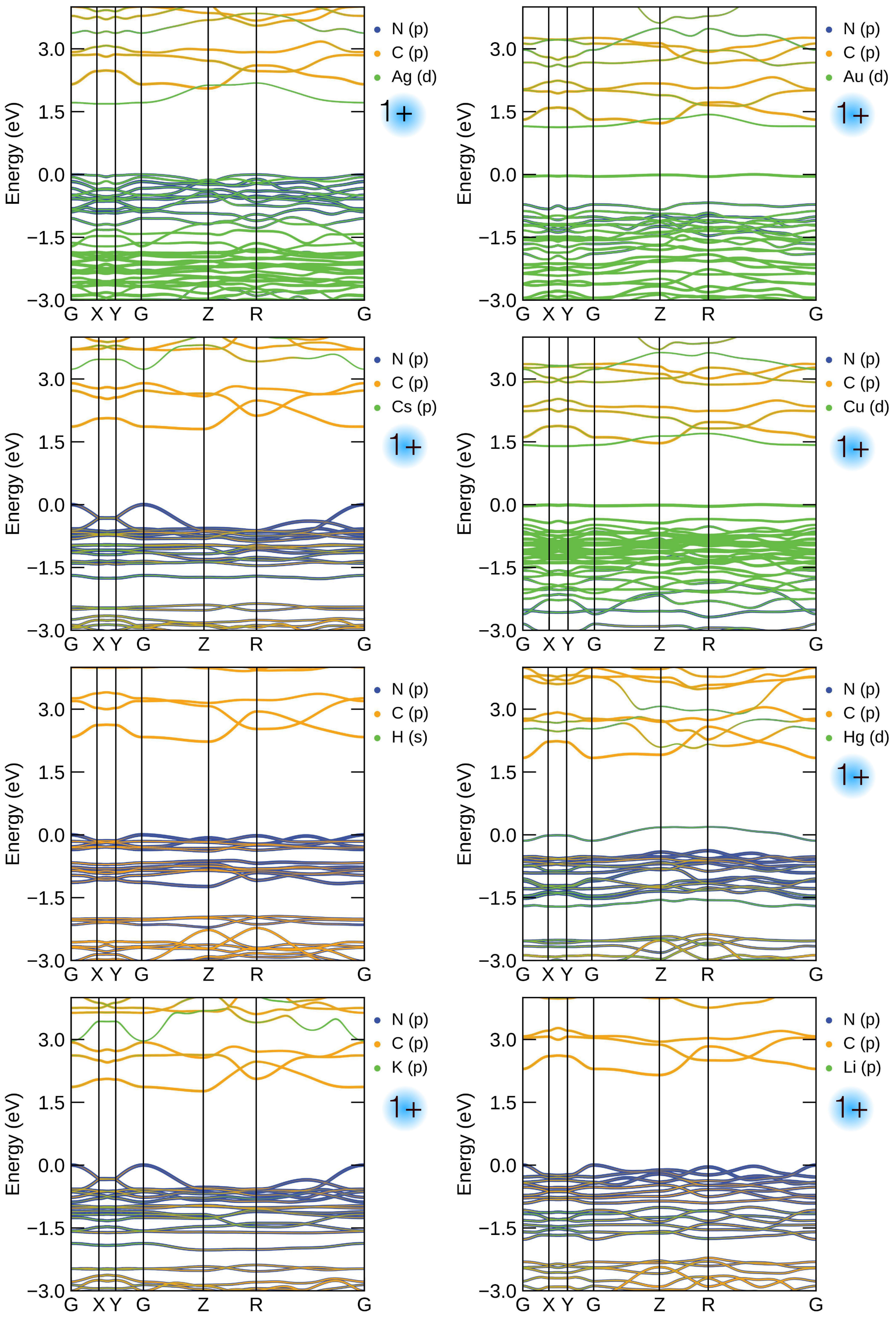
Energy (eV)
3.0
1.5
0.0
−1.5
−3.0
G X Y G Z R G
N (p)
C (p)
Ag (d)
Au (d)
Cs (p)
Cu (d)
H (s)
Hg (d)
K (p)
Li (p)
1+

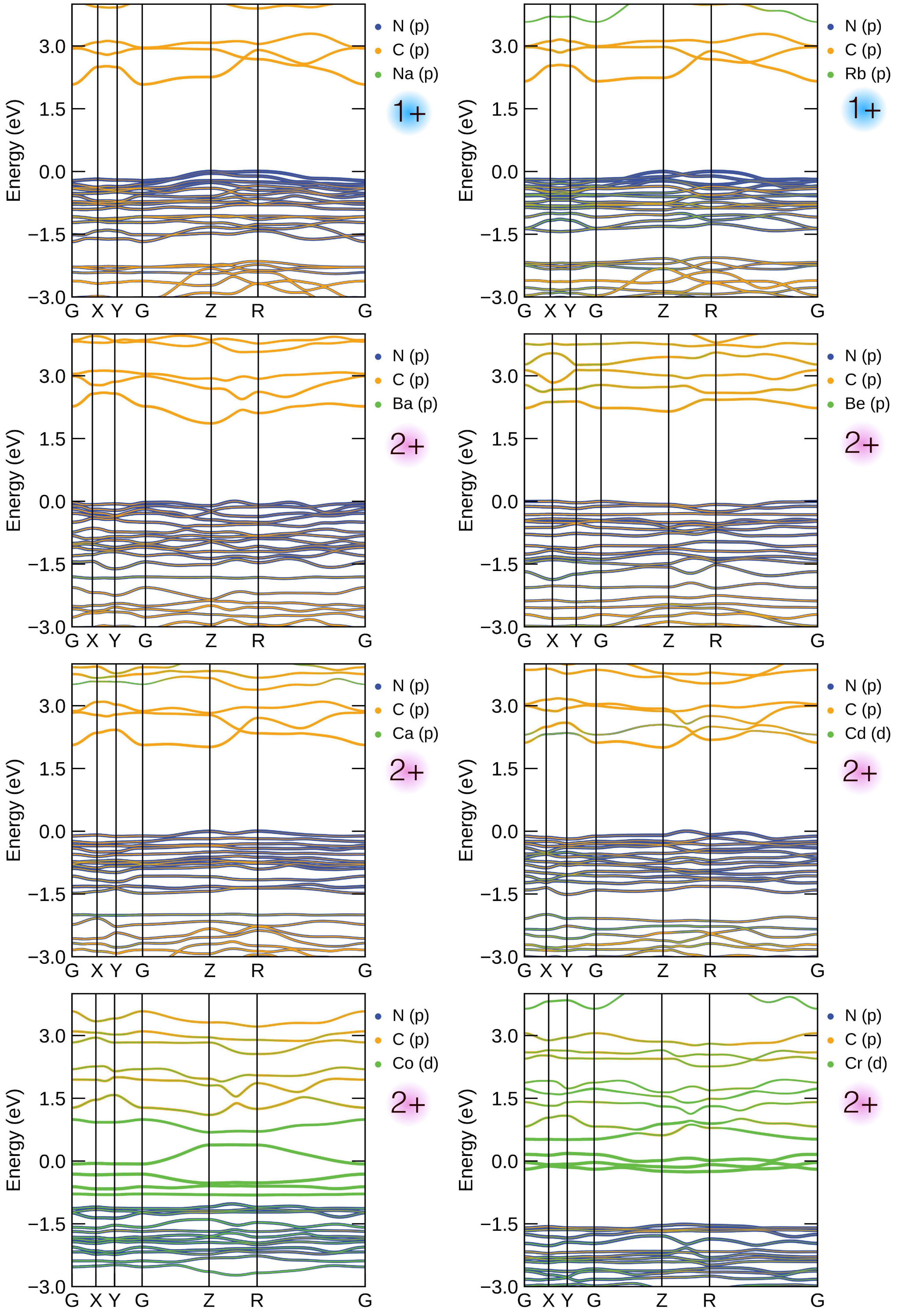
Energy (eV)
3.0
1.5
0.0
−1.5
−3.0
G X Y G Z R G
N (p)
C (p)
Na (p)
1+
Rb (p)
1+
Ba (p)
2+
Be (p)
2+
Ca (p)
2+
Cd (d)
2+
Co (d)
2+
Cr (d)
2+

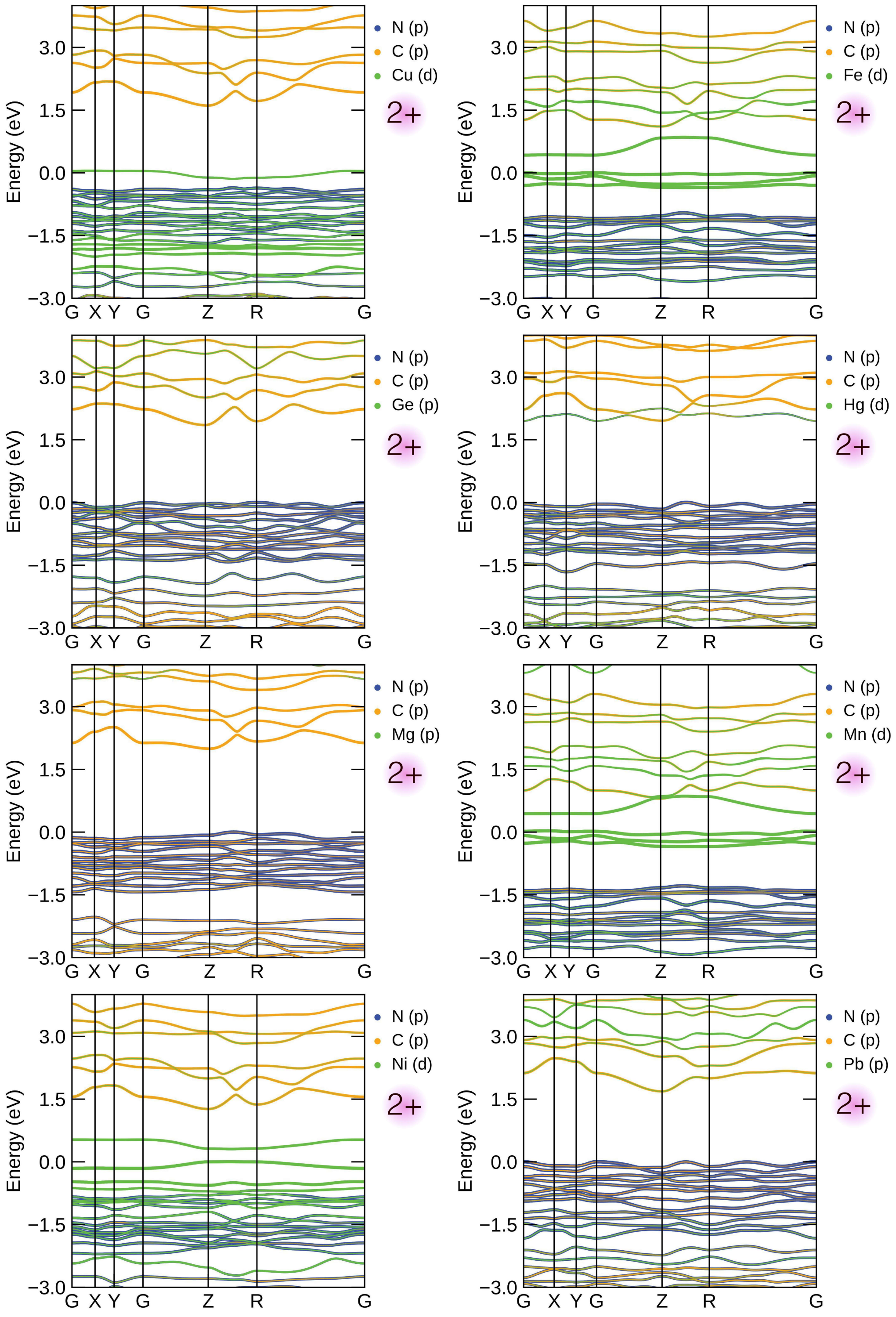
Energy (eV)
3.0
1.5
0.0
−1.5
−3.0
G X Y G Z R G
N (p)
C (p)
Cu (d)
Fe (d)
Ge (p)
Hg (d)
Mg (p)
Mn (d)
Ni (d)
Pb (p)
2+

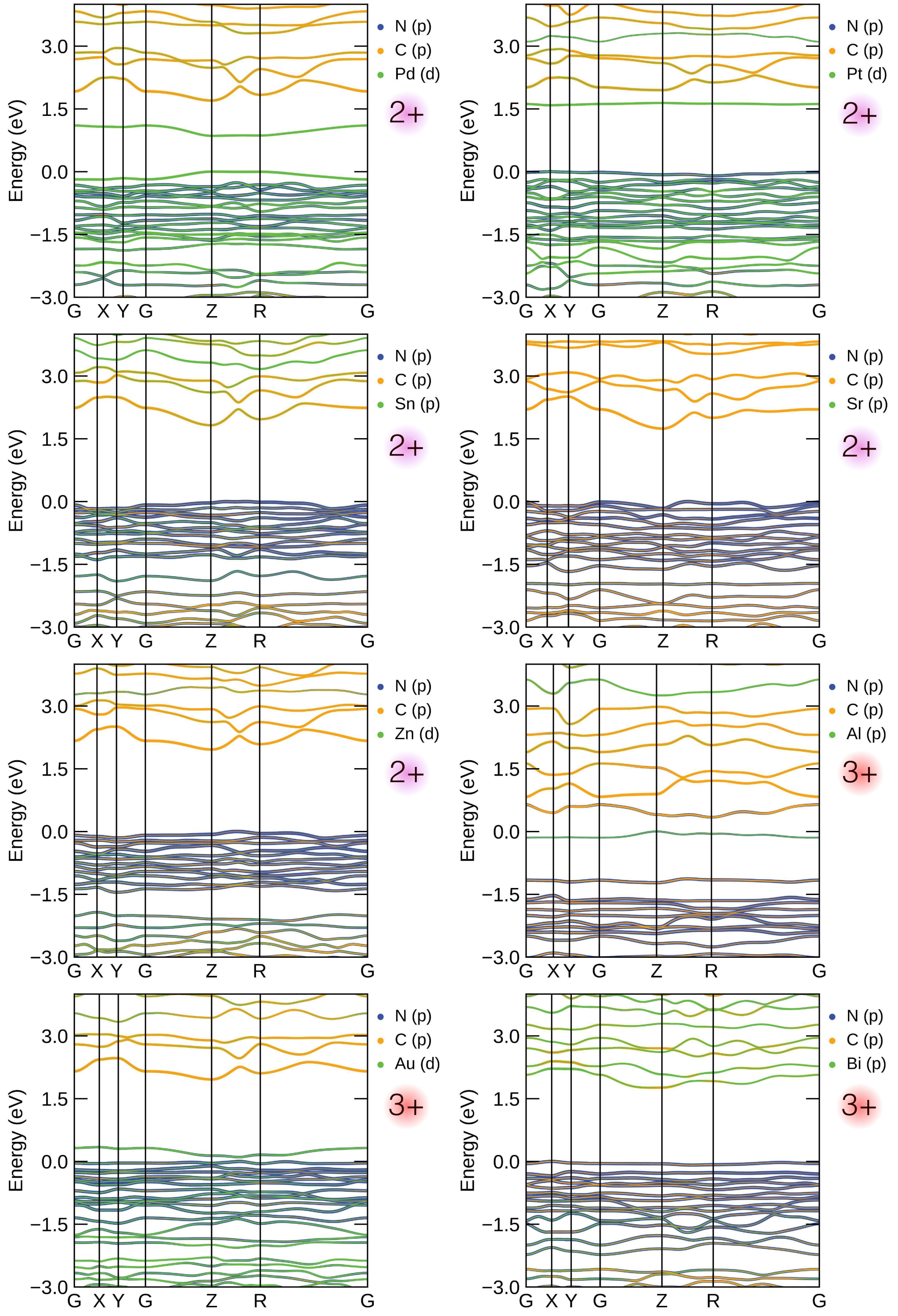
Energy (eV)
3.0
1.5
0.0
−1.5
−3.0
G X Y G Z R G
N (p)
C (p)
Pd (d)
2+
N (p)
C (p)
Pt (d)
2+
N (p)
C (p)
Sn (p)
2+
N (p)
C (p)
Sr (p)
2+
N (p)
C (p)
Zn (d)
2+
N (p)
C (p)
Al (p)
3+
N (p)
C (p)
Au (d)
3+
N (p)
C (p)
Bi (p)
3+

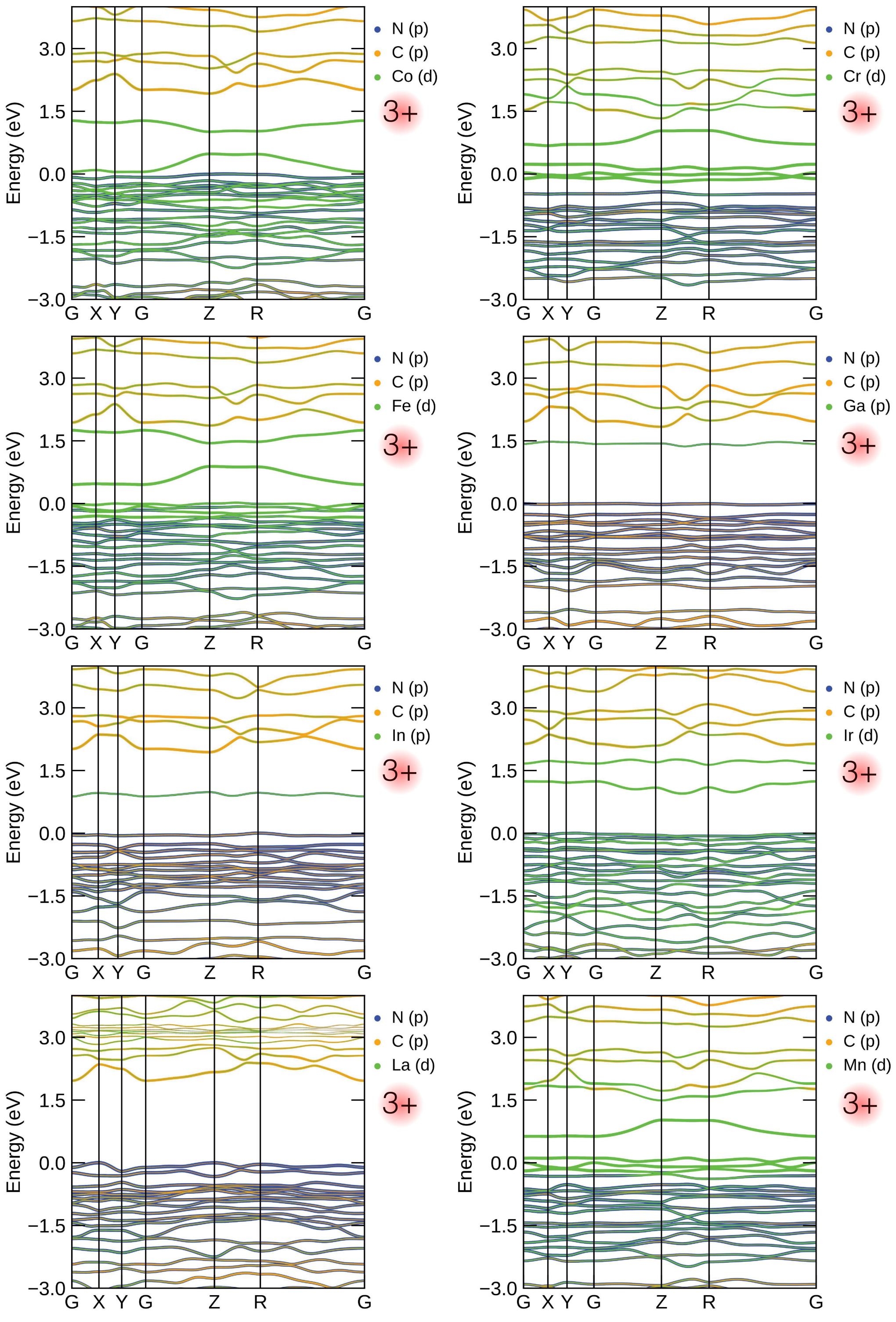
Energy (eV)
3.0
1.5
0.0
−1.5
−3.0
G X Y G Z R G
N (p)
C (p)
Co (d)
3+
N (p)
C (p)
Cr (d)
3+
N (p)
C (p)
Fe (d)
3+
N (p)
C (p)
Ga (p)
3+
N (p)
C (p)
In (p)
3+
N (p)
C (p)
Ir (d)
3+
N (p)
C (p)
La (d)
3+
N (p)
C (p)
Mn (d)
3+

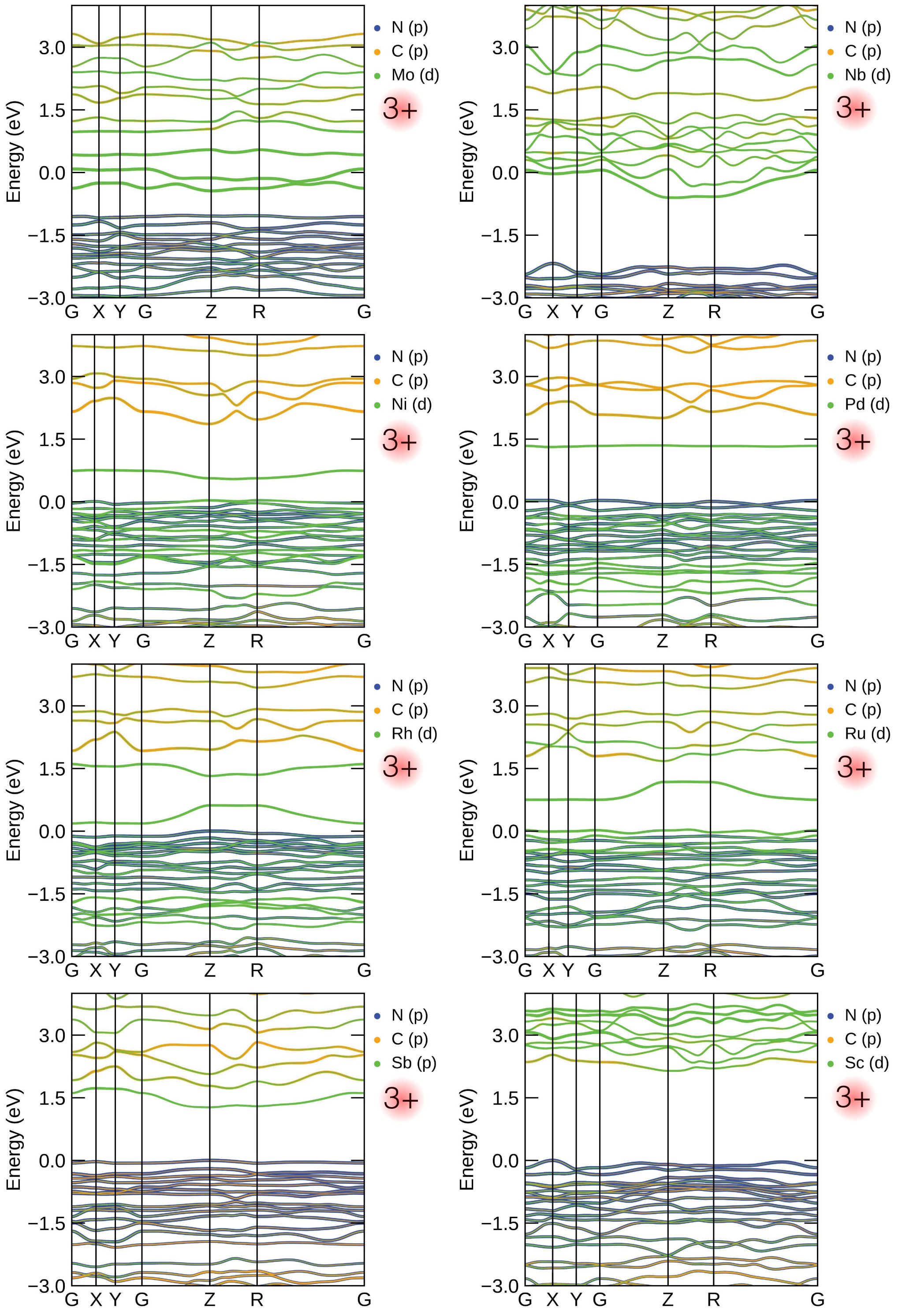
N (p)
C (p)
Mo (d)
Nb (d)
Ni (d)
Pd (d)
Rh (d)
Ru (d)
Sb (p)
Sc (d)
3+
Energy (eV)
G X Y G Z R G
3.0
1.5
0.0
−1.5
−3.0

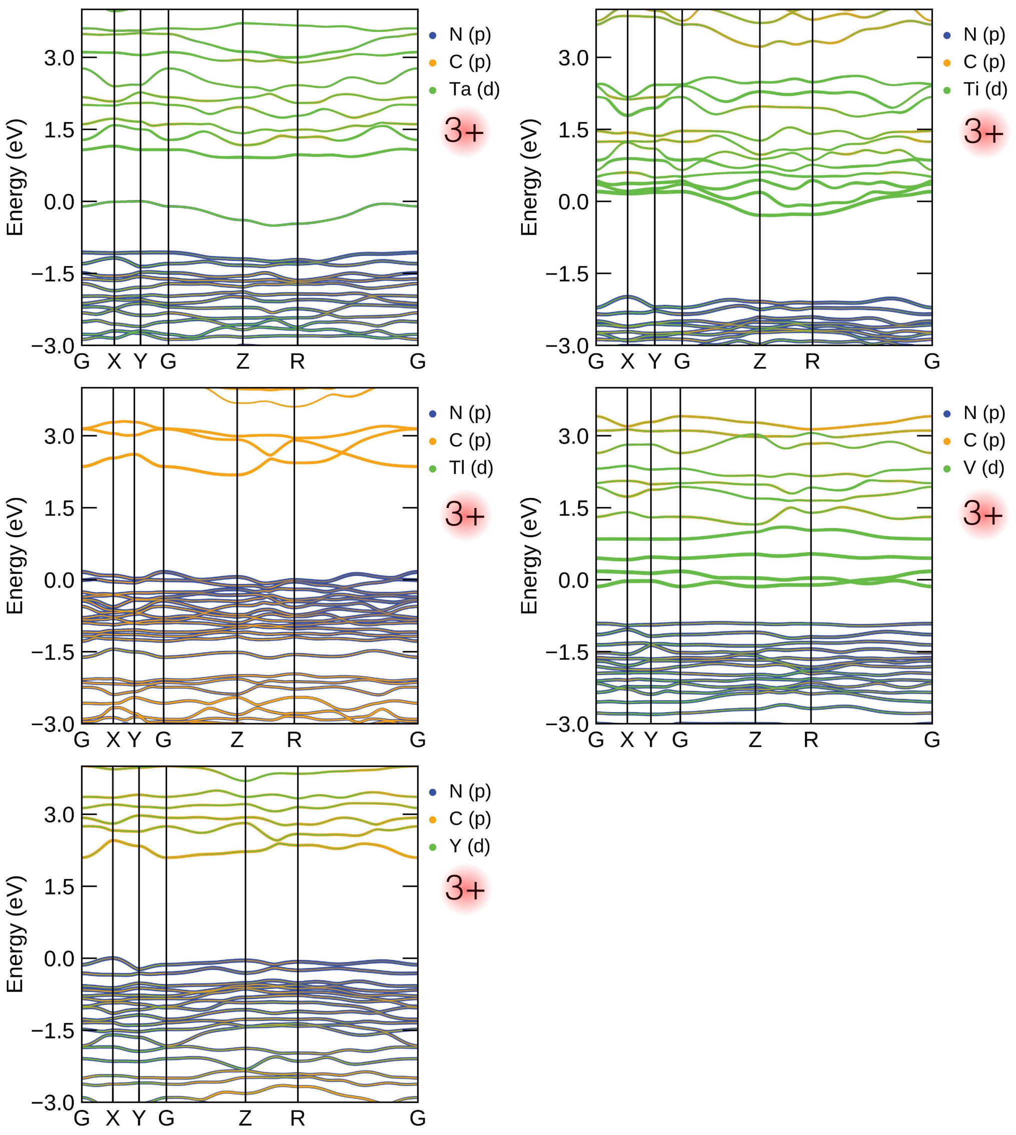

Fig. S8: Projected band structure

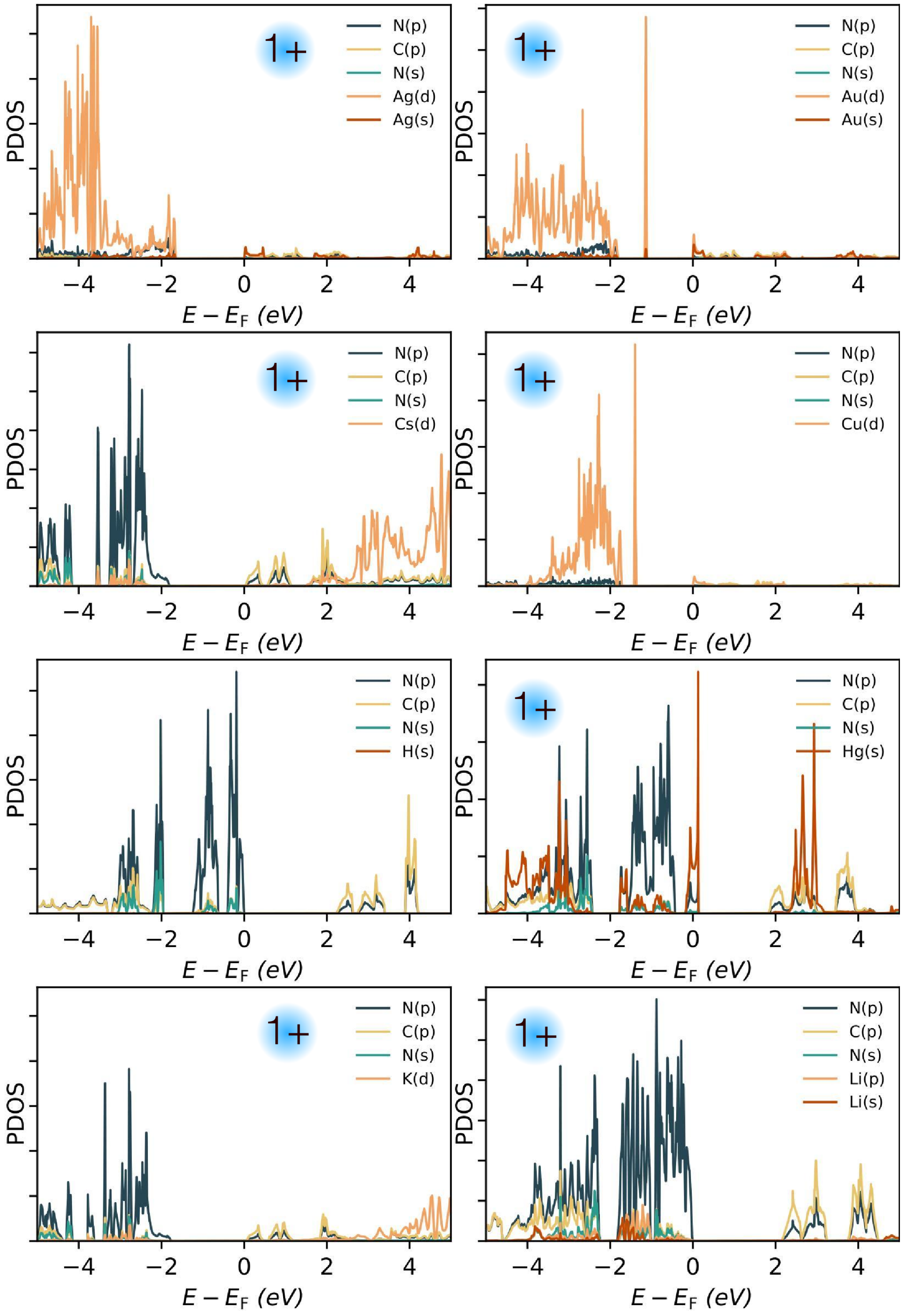
1+
N(p)
C(p)
N(s)
Ag(d)
Ag(s)
PDOS
E − E_F (eV)
1+
N(p)
C(p)
N(s)
Au(d)
Au(s)
PDOS
E − E_F (eV)
1+
N(p)
C(p)
N(s)
Cs(d)
PDOS
E − E_F (eV)
1+
N(p)
C(p)
N(s)
Cu(d)
PDOS
E − E_F (eV)
N(p)
C(p)
N(s)
H(s)
PDOS
E − E_F (eV)
1+
N(p)
C(p)
N(s)
Hg(s)
PDOS
E − E_F (eV)
1+
N(p)
C(p)
N(s)
K(d)
PDOS
E − E_F (eV)
1+
N(p)
C(p)
N(s)
Li(p)
Li(s)
PDOS
E − E_F (eV)
−4
−2
0
2
4

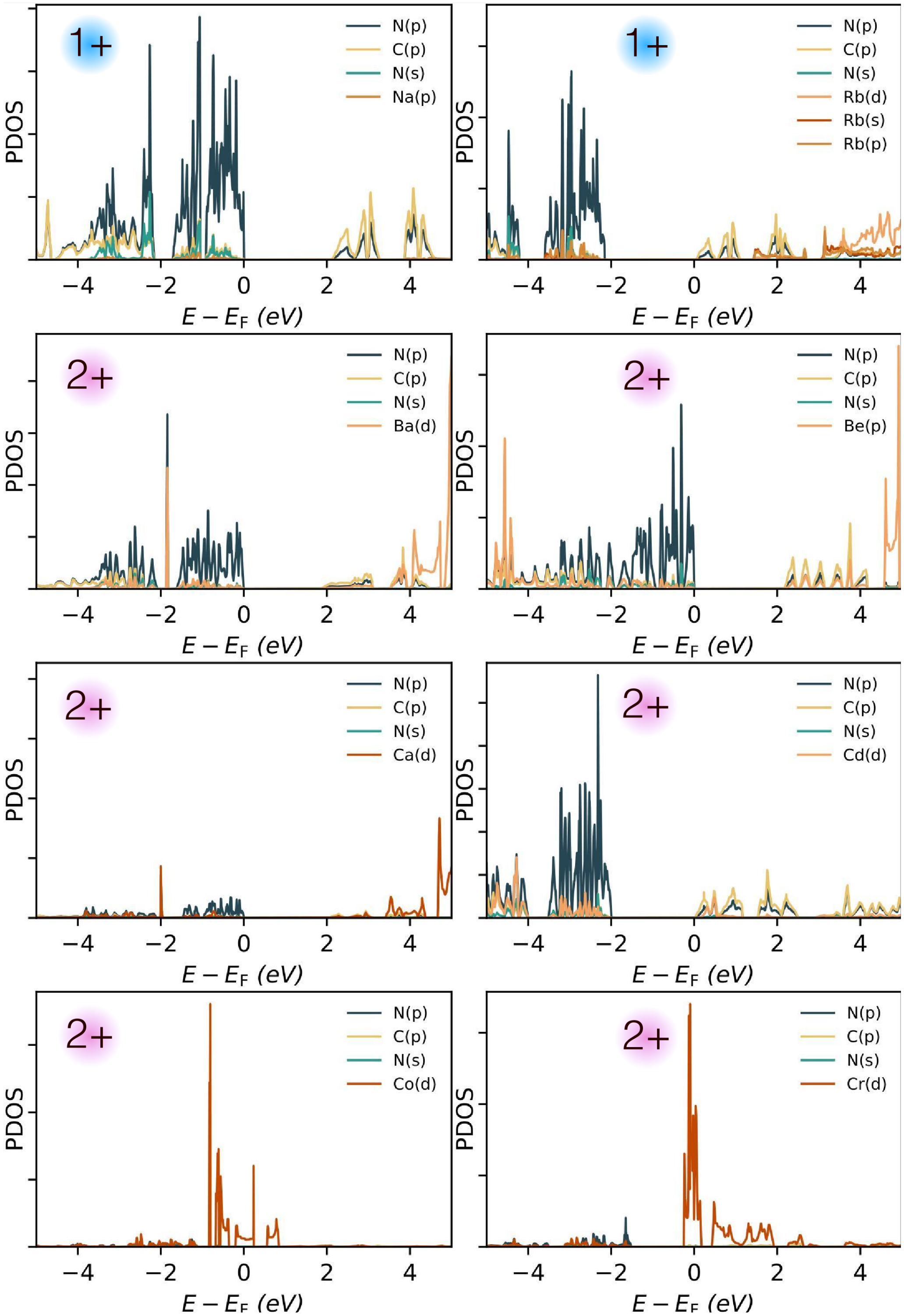
1+
N(p)
C(p)
N(s)
Na(p)
Rb(d)
Rb(s)
Rb(p)
2+
Ba(d)
Be(p)
Ca(d)
Cd(d)
Co(d)
Cr(d)
PDOS
$E - E_F$ (eV)

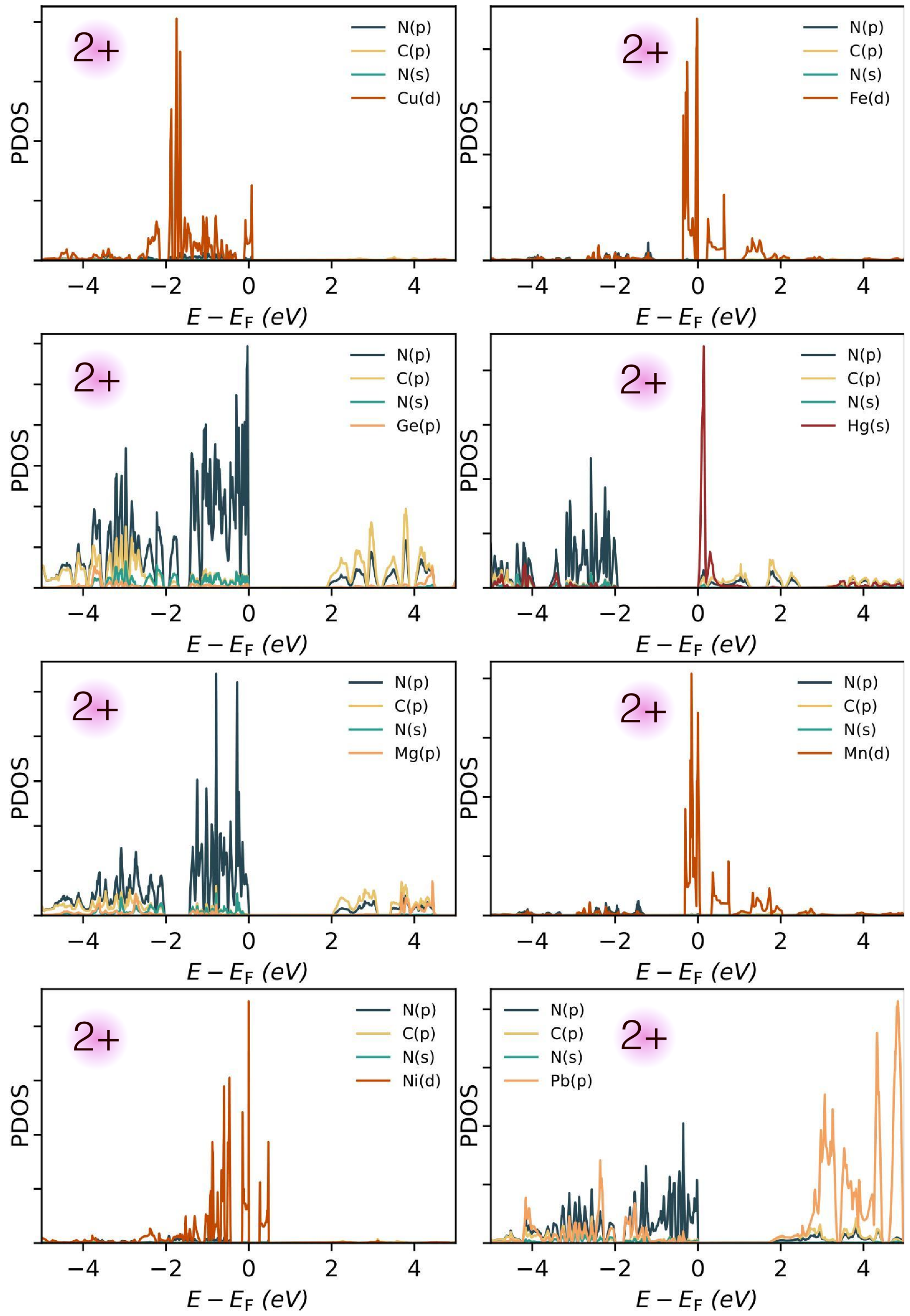
2+
N(p)
C(p)
N(s)
Cu(d)
PDOS
−4 −2 0 2 4
E − E_F (eV)
2+
N(p)
C(p)
N(s)
Fe(d)
PDOS
−4 −2 0 2 4
E − E_F (eV)
2+
N(p)
C(p)
N(s)
Ge(p)
PDOS
−4 −2 0 2 4
E − E_F (eV)
2+
N(p)
C(p)
N(s)
Hg(s)
PDOS
−4 −2 0 2 4
E − E_F (eV)
2+
N(p)
C(p)
N(s)
Mg(p)
PDOS
−4 −2 0 2 4
E − E_F (eV)
2+
N(p)
C(p)
N(s)
Mn(d)
PDOS
−4 −2 0 2 4
E − E_F (eV)
2+
N(p)
C(p)
N(s)
Ni(d)
PDOS
−4 −2 0 2 4
E − E_F (eV)
N(p)
C(p)
N(s)
Pb(p)
2+
PDOS
−4 −2 0 2 4
E − E_F (eV)

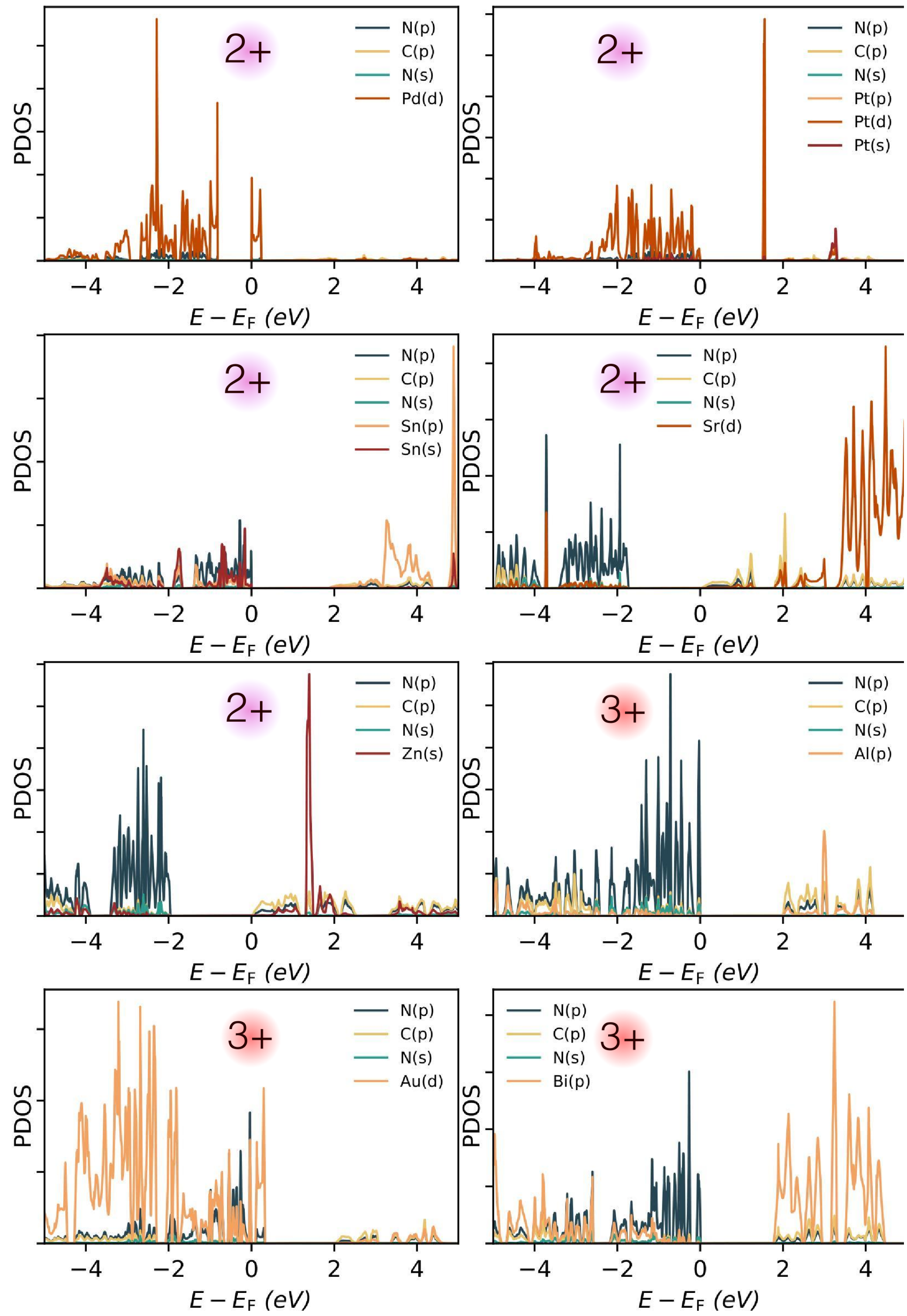
2+
N(p)
C(p)
N(s)
Pd(d)
PDOS
−4 −2 0 2 4
E − E_F (eV)
2+
N(p)
C(p)
N(s)
Pt(p)
Pt(d)
Pt(s)
2+
N(p)
C(p)
N(s)
Sn(p)
Sn(s)
2+
N(p)
C(p)
N(s)
Sr(d)
2+
N(p)
C(p)
N(s)
Zn(s)
3+
N(p)
C(p)
N(s)
Al(p)
3+
N(p)
C(p)
N(s)
Au(d)
3+
N(p)
C(p)
N(s)
Bi(p)

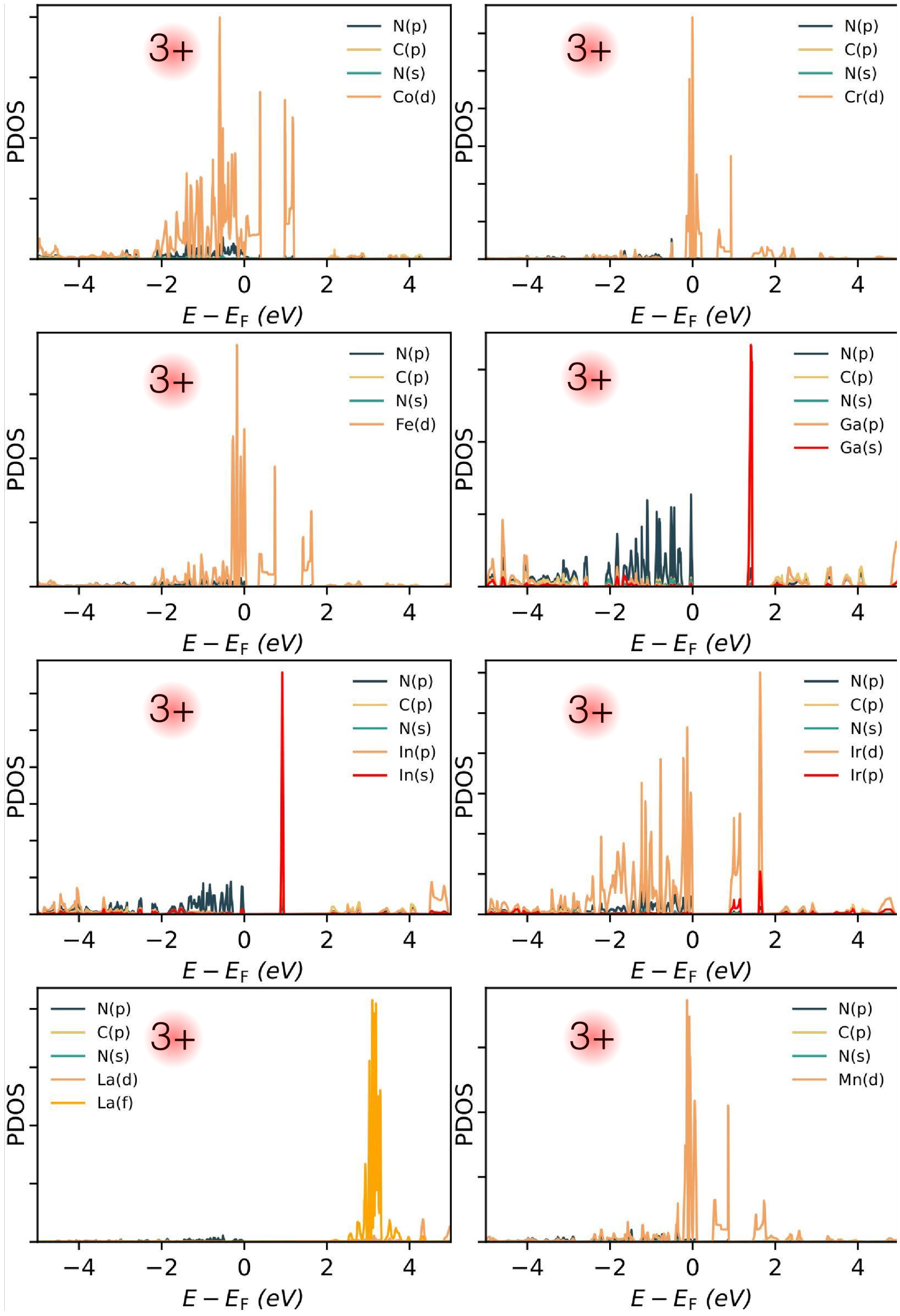
3+
N(p)
C(p)
N(s)
Co(d)
Cr(d)
Fe(d)
Ga(p)
Ga(s)
In(p)
In(s)
Ir(d)
Ir(p)
La(d)
La(f)
Mn(d)
PDOS
−4
−2
0
2
4
$E - E_F$ (eV)

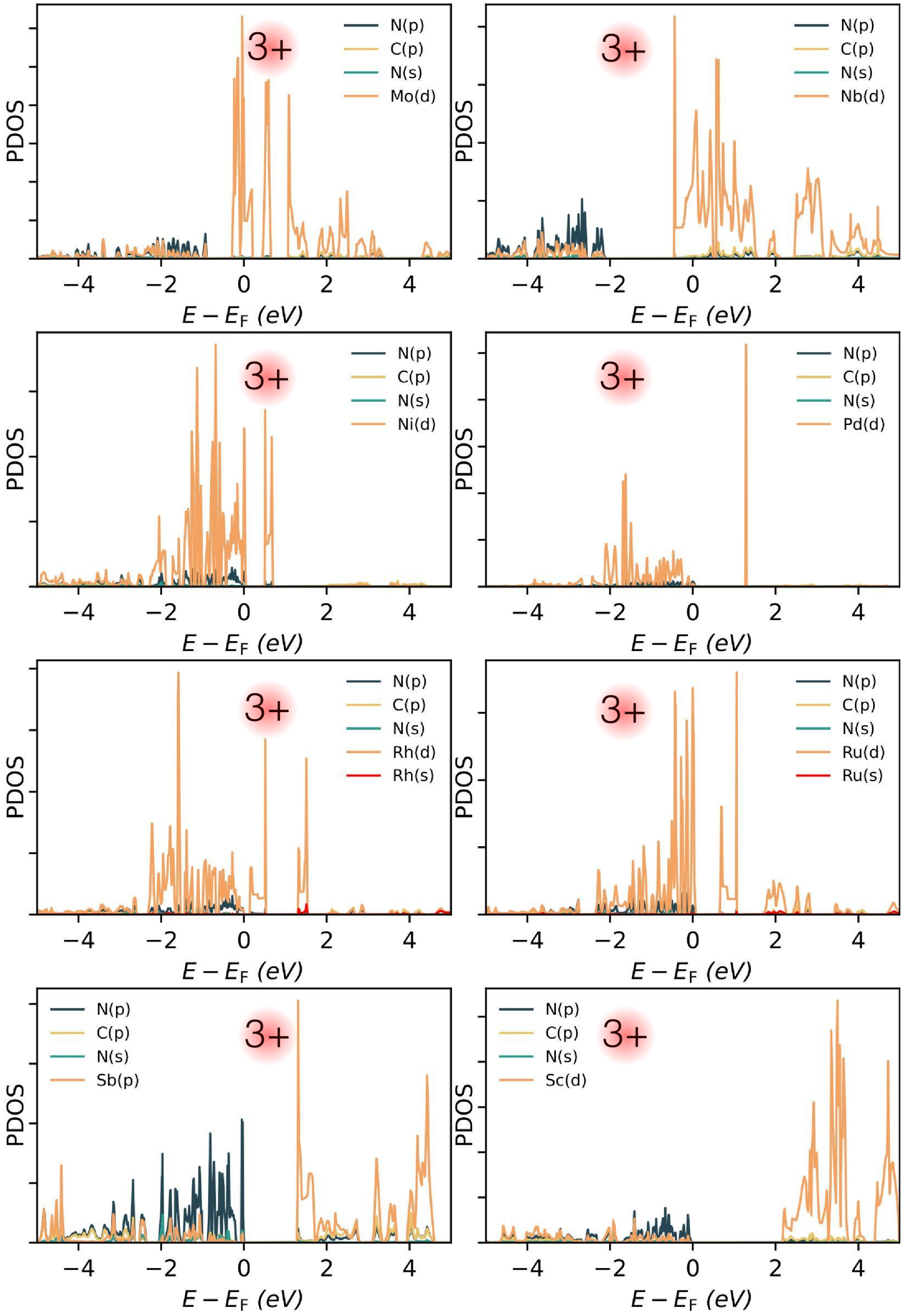
3+
N(p)
C(p)
N(s)
Mo(d)
Nb(d)
Ni(d)
Pd(d)
Rh(d)
Rh(s)
Ru(d)
Ru(s)
Sb(p)
Sc(d)
PDOS
−4
−2
0
2
4
$E - E_F$ (eV)

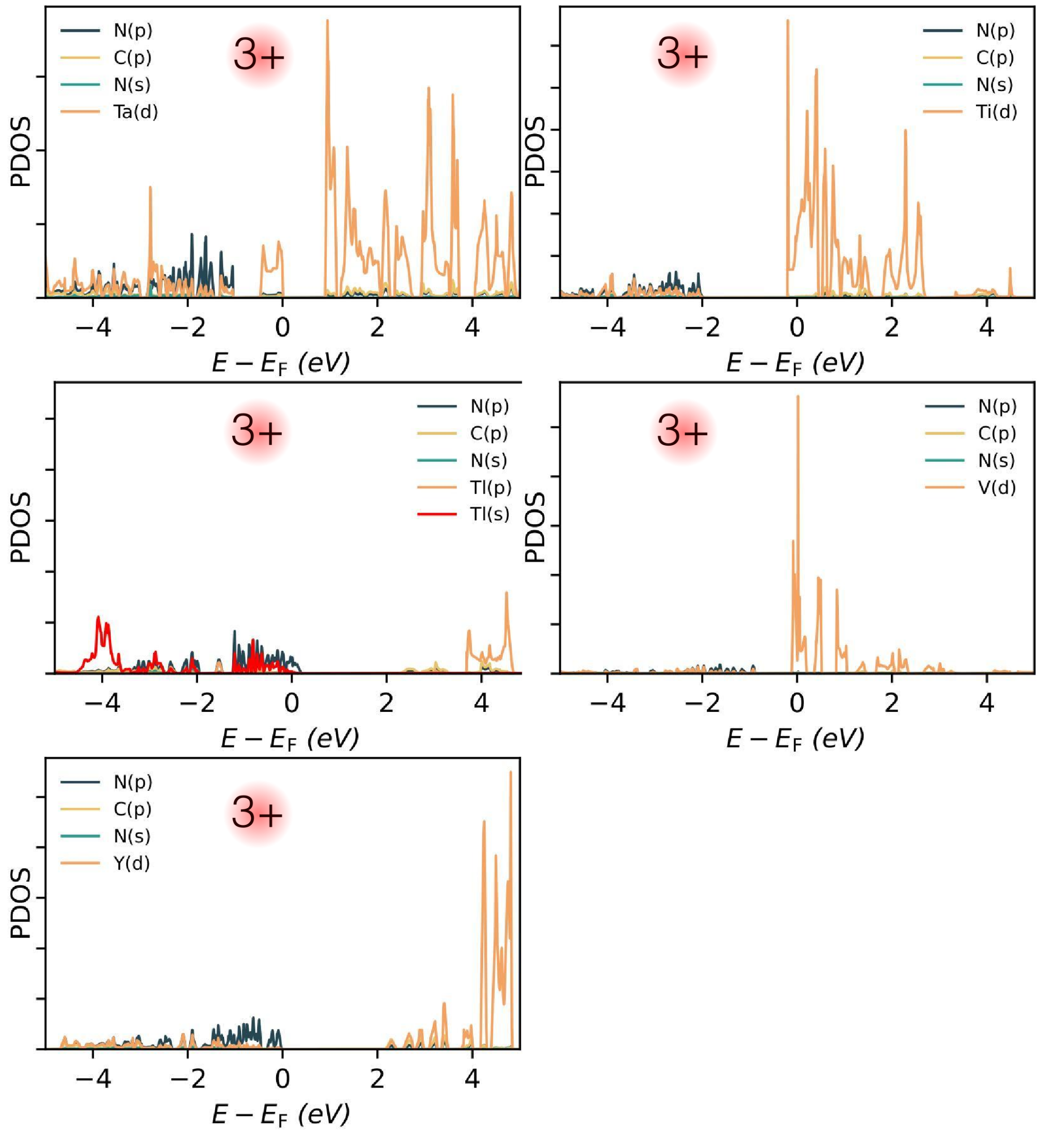

Fig. S9: Projected density of state

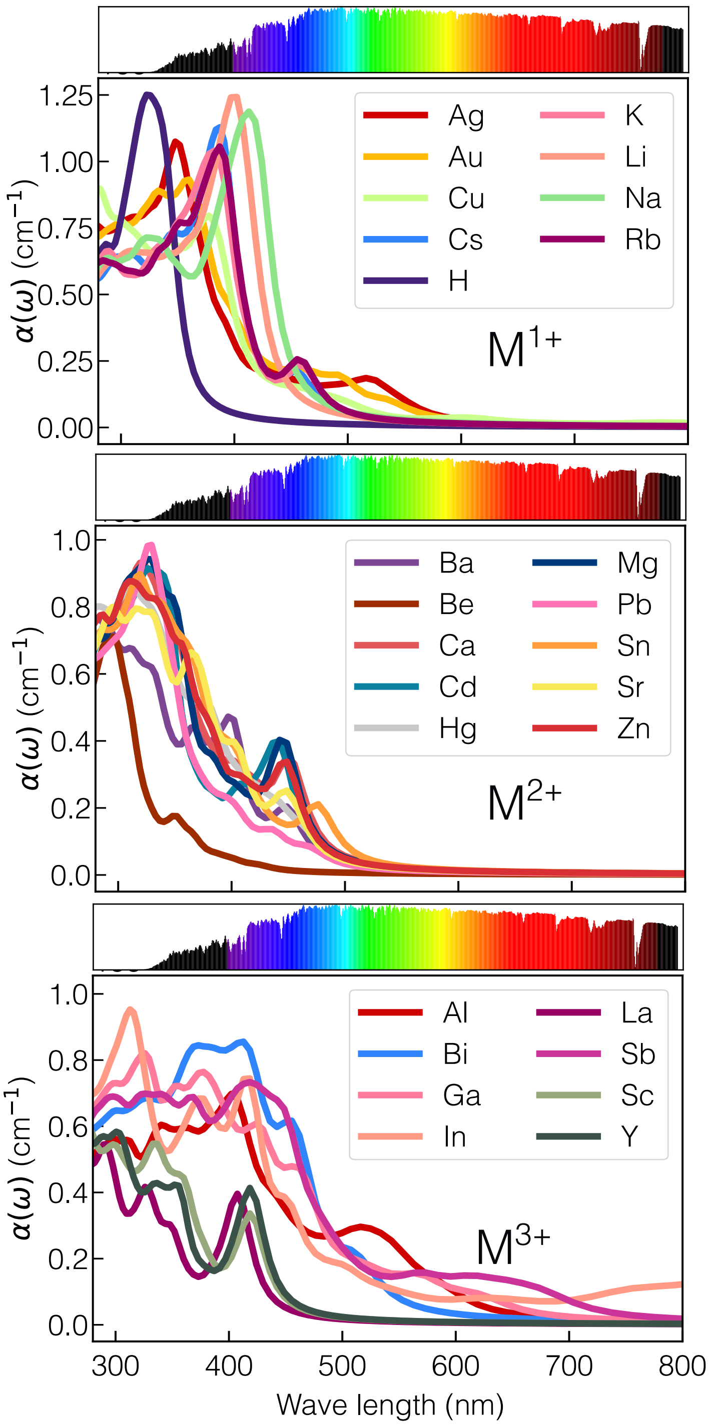

Fig. S10: Absorption coefficient with GW/BSE method for cations, M^{1+}, M^{2+}, and M^{3+}.

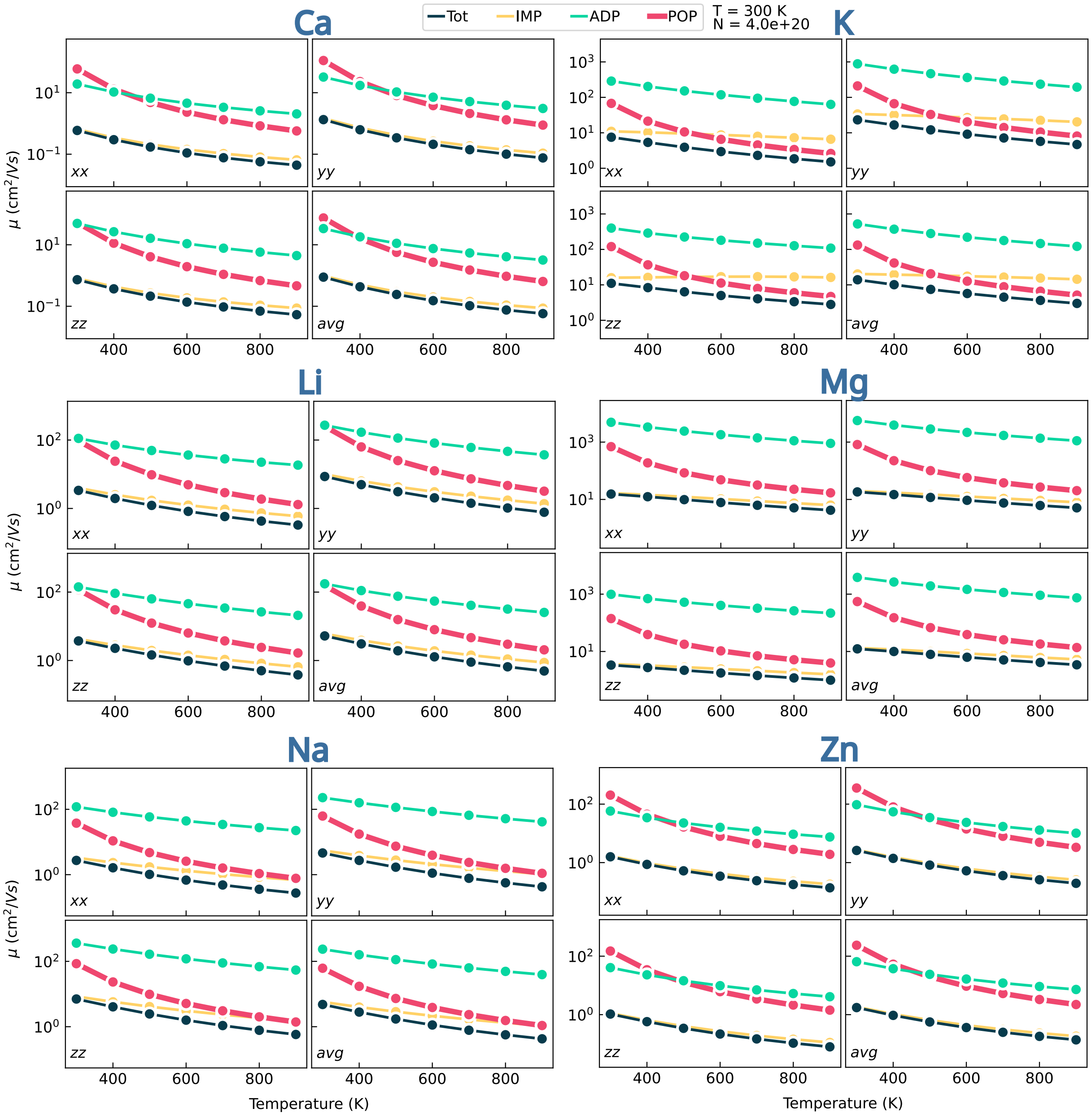

Fig. S 11: Carrier mobility (μ) for electron doped $4.0\,\mathrm{e}20\,\mathrm{cm}$, at 300 K, and temperature-dependent in acoustic phonon (AP), polar optical phonon (POP), and ionized impurity(IMP)

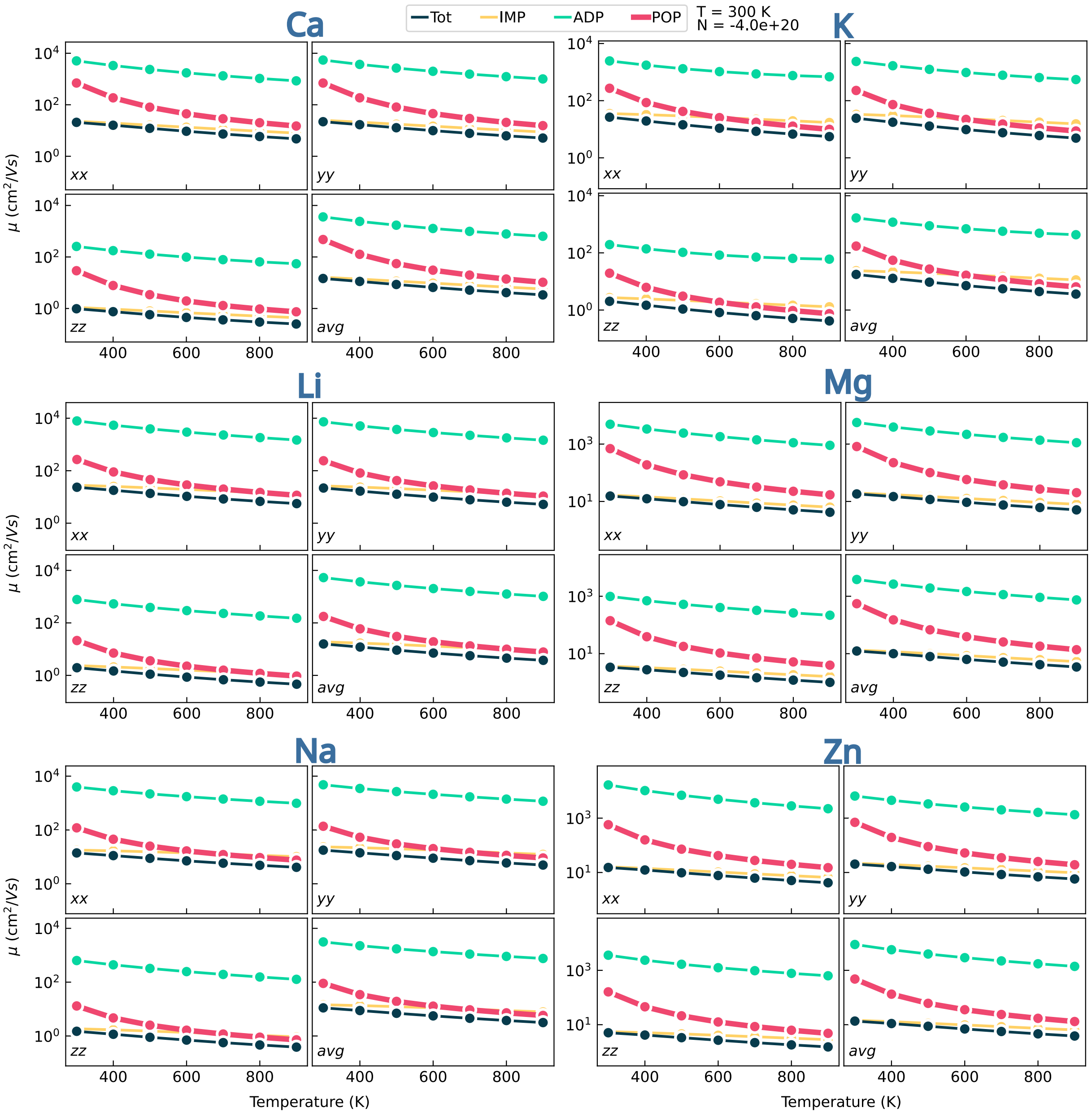

Fig. S12: Carrier mobility (μ) for hole doped -4.0 e20 cm at 300 K, and temperature-dependent in acoustic phonon (AP), polar optical phonon (POP), and ionized impurity(IMP)
